# The Arabidopsis ARF3-AIP1/2-SAP18 module specifies the root stem cell niche in response to auxin

**DOI:** 10.1093/plcell/koag108

**Published:** 2026-04-10

**Authors:** Zhi Juan Cheng, Meng Ru Zhang, Huan Kai Zhang, Xiao Li Chu, Bing Zhen Li, Miao Miao Zhang, Jia Yang Li, Wan Chen Dong, De Hao Wang, Wen Qi Xin, Xin Lin Han, Cai Yu Yu, Zhi Wei Wang, Xiao Hang Zhang, Jiong Hui Liu, Xian Sheng Zhang, Ya Lin Sang

**Affiliations:** College of Life Sciences, College of Forestry,State Key Laboratory of Wheat Improvement, Key Laboratory for Warm Temperate Forest Ecosystem Conservation and Restoration of National Forestry and Grassland Administration, Shandong Agricultural University, Taian, Shandong 271018, China; College of Life Sciences, College of Forestry,State Key Laboratory of Wheat Improvement, Key Laboratory for Warm Temperate Forest Ecosystem Conservation and Restoration of National Forestry and Grassland Administration, Shandong Agricultural University, Taian, Shandong 271018, China; College of Life Sciences, College of Forestry,State Key Laboratory of Wheat Improvement, Key Laboratory for Warm Temperate Forest Ecosystem Conservation and Restoration of National Forestry and Grassland Administration, Shandong Agricultural University, Taian, Shandong 271018, China; College of Life Sciences, Zaozhuang University, Zaozhuang 277160, China; College of Life Sciences, College of Forestry,State Key Laboratory of Wheat Improvement, Key Laboratory for Warm Temperate Forest Ecosystem Conservation and Restoration of National Forestry and Grassland Administration, Shandong Agricultural University, Taian, Shandong 271018, China; College of Life Sciences, College of Forestry,State Key Laboratory of Wheat Improvement, Key Laboratory for Warm Temperate Forest Ecosystem Conservation and Restoration of National Forestry and Grassland Administration, Shandong Agricultural University, Taian, Shandong 271018, China; College of Life Sciences, College of Forestry,State Key Laboratory of Wheat Improvement, Key Laboratory for Warm Temperate Forest Ecosystem Conservation and Restoration of National Forestry and Grassland Administration, Shandong Agricultural University, Taian, Shandong 271018, China; College of Life Sciences, College of Forestry,State Key Laboratory of Wheat Improvement, Key Laboratory for Warm Temperate Forest Ecosystem Conservation and Restoration of National Forestry and Grassland Administration, Shandong Agricultural University, Taian, Shandong 271018, China; College of Life Sciences, College of Forestry,State Key Laboratory of Wheat Improvement, Key Laboratory for Warm Temperate Forest Ecosystem Conservation and Restoration of National Forestry and Grassland Administration, Shandong Agricultural University, Taian, Shandong 271018, China; College of Life Sciences, College of Forestry,State Key Laboratory of Wheat Improvement, Key Laboratory for Warm Temperate Forest Ecosystem Conservation and Restoration of National Forestry and Grassland Administration, Shandong Agricultural University, Taian, Shandong 271018, China; College of Life Sciences, College of Forestry,State Key Laboratory of Wheat Improvement, Key Laboratory for Warm Temperate Forest Ecosystem Conservation and Restoration of National Forestry and Grassland Administration, Shandong Agricultural University, Taian, Shandong 271018, China; College of Life Sciences, College of Forestry,State Key Laboratory of Wheat Improvement, Key Laboratory for Warm Temperate Forest Ecosystem Conservation and Restoration of National Forestry and Grassland Administration, Shandong Agricultural University, Taian, Shandong 271018, China; College of Life Sciences, College of Forestry,State Key Laboratory of Wheat Improvement, Key Laboratory for Warm Temperate Forest Ecosystem Conservation and Restoration of National Forestry and Grassland Administration, Shandong Agricultural University, Taian, Shandong 271018, China; College of Life Sciences, Zaozhuang University, Zaozhuang 277160, China; College of Life Sciences, College of Forestry,State Key Laboratory of Wheat Improvement, Key Laboratory for Warm Temperate Forest Ecosystem Conservation and Restoration of National Forestry and Grassland Administration, Shandong Agricultural University, Taian, Shandong 271018, China; College of Life Sciences, College of Forestry,State Key Laboratory of Wheat Improvement, Key Laboratory for Warm Temperate Forest Ecosystem Conservation and Restoration of National Forestry and Grassland Administration, Shandong Agricultural University, Taian, Shandong 271018, China; College of Life Sciences, College of Forestry,State Key Laboratory of Wheat Improvement, Key Laboratory for Warm Temperate Forest Ecosystem Conservation and Restoration of National Forestry and Grassland Administration, Shandong Agricultural University, Taian, Shandong 271018, China; College of Life Sciences, College of Forestry,State Key Laboratory of Wheat Improvement, Key Laboratory for Warm Temperate Forest Ecosystem Conservation and Restoration of National Forestry and Grassland Administration, Shandong Agricultural University, Taian, Shandong 271018, China; College of Life Sciences, College of Forestry,State Key Laboratory of Wheat Improvement, Key Laboratory for Warm Temperate Forest Ecosystem Conservation and Restoration of National Forestry and Grassland Administration, Shandong Agricultural University, Taian, Shandong 271018, China

## Abstract

In the root apical meristem, the stem cell niche (SCN) comprises a mitotically inactive quiescent center (QC) and adjacent, mitotically active stem cells that divide to form root tissues. Auxin dynamics are essential for specification and maintenance of the root SCN; however, the underlying mechanisms remain to be explored. Here, we report that Arabidopsis (*Arabidopsis thaliana*) AUXIN RESPONSE FACTOR3 (ARF3), ARF3-INTERACTING PROTEIN1/2 (AIP1/2), and SIN3-ASSOCIATED POLYPEPTIDE OF 18 KDA (SAP18) form a protein complex that specifies root SCN cell fate in response to auxin level. In cells proximal to the QC, the ARF3-AIP1/2-SAP18 complex bound the *WOX5* promoter and restricted *WOX5* expression to the QC by decreasing H3 histone acetylation, thereby maintaining the SCN. Disrupting the ARF3-AIP1/2-SAP18 complex via mutation or exposure to excessive amounts of auxin resulted in proximal and lateral expansion of *WOX5* expression and inhibited root elongation by repressing cell division. During de novo specification of the SCN in lateral root primordia or regenerating root tips, accumulated auxin caused the ARF3-AIP1/2-SAP18 complex to dissociate, allowing the induction of *WOX5* expression. In the reestablished meristem, the ARF3-AIP1/2-SAP18 complex confines *WOX5* expression to the newly formed QC. Our findings provide insights into the roles of auxin dynamics in determining root SCN.

## Teaser

The ARF3-AIP1/2-SAP18 complex confines *WOX5* expression by modulating H3 histone acetylation in response to auxin level and thus regulates the maintenance and regeneration of root stem cell niche.

## Introduction

Unlike their animal counterparts, land plants possess the ability to continuously generate new tissues and organs during their post-embryonic development (Wolters and Jürgens 2009; Ko and Helariutta 2017). This is enabled by activity of the stem cell niches (SCNs) reside in the apical meristems at the opposite poles of the primary axis (Scheres 2007). The SCN in the root apical meristem encompasses the quiescent center (QC) and an abutting layer of stem cells. Depending on their position, these stem cells generate different types of root cells (Motte et al. 2019). Stem cells localized proximal to the QC give rise to the cortex, endodermis, and stele, while those located lateral to the QC produce the epidermis and lateral root cap. Distal stem cells generate the columella. The QC, which comprises a set of mitotically inactive cells, serves as an organizer and reservoir of the stem cell. Laser ablation of QC cells ceases division and initiates differentiation of surrounding stem cells (van den Berg et al. 1997). After injury, QC cells divide to replenish damaged stem cells (Heyman et al. 2013; Zhou et al. 2019).

Root SCN homeostasis is controlled by a complex network of regulatory genes (Pardal and Heidstra 2021). Of these, *WUSCHEL-RELATED HOMEOBOX 5* (*WOX5*), which is specifically expressed in the QC and encodes a homeodomain transcription factor, plays essential roles in regulating stem cell maintenance and QC identity (Zhang et al. 2025). In the QC, WOX5 represses cell division by directly suppressing *cyclin D3;3* (*CYCD3;3*) expression via a cell-autonomous pathway, which involves the direct interaction with BRASSINOSTEROIDS AT VASCULAR AND ORGANIZING CENTER and PLETHORA3 (Forzani et al. 2014; Betegón-Putze et al. 2021; Burkart et al. 2022). In the columella stem cell, WOX5 maintains the undifferentiated state by directly downregulating the transcription of the differentiation-related gene *CYCLING DOF FACTOR 4* in a non–cell-autonomous manner (Pi et al. 2015). The specific *WOX5* expression pattern is required for proper SCN behavior (Strotmann and Stahl 2021). Previous studies have shown that *WOX5* expression is induced by the PLETHORA (PLT)-the teosinte-branched cycloidea PCNA (TCP)-SCARECROW (SCR) complex, and restricted into the QC by REPRESSOR OF WUSCHEL 1 and AUXIN RESPONSE FACTOR 10/16 (Han et al. 2008; Ding and Friml 2010; Zhang et al. 2015; Shimotohno et al. 2018). Moreover, the subnuclear partitioning and stabilization of WOX5 also influence the maintenance of the columella stem cell (Burkart et al. 2022; Cui et al. 2024).

Phytohormone auxin plays critical roles in determining *WOX5* expression and root SCN specification (Roychoudhry and Kepinski 2022). In the distal root tip, the polar transport and local biosynthesis create a graded auxin accumulation that culminates in the QC (Sabatini et al. 1999; Petersson et al. 2009). *WOX5* expression and QC identity overlap and depend on the auxin maximum, which is required for SCN maintenance. Lateral shift of auxin maximum caused by inhibited polar transport leads to concomitant transfer of QC and columella initial identities (Sabatini et al. 1999). Exogenous auxin treatment expands *WOX5* expression domain, whereas decreases in auxin levels downregulate *WOX5* expression and lead to QC cell division (Friml et al. 2002; Goh et al. 2012; Mähönen et al. 2014). During lateral root formation and root-tip regeneration, the root SCN is de novo respecified. In these processes, *WOX5* expression is initially induced in broad areas and then gradually confined to the new QC (Benková et al. 2003; Efroni et al. 2016; Shimotohno et al. 2018). This expression pattern is correlated with the accumulation of indole 3-acetic acid (IAA) during the establishment of lateral root primordia. Moreover, after root-tip excision, local auxin biosynthesis near the cut site is required for *WOX5* expression and the subsequent re-establishment of the SCN (Sena et al. 2009; Matosevich et al. 2020). In spite of the tight relation between auxin dynamics and *WOX5* expression and SCN specification, the underlying mechanisms remain to be elucidated.

Auxin-responsive gene transcription is mediated by AUXIN RESPONSE FACTOR (ARF) family transcription factors (Weijers and Wagner 2016; Roosjen et al. 2018). In canonical auxin signaling, Auxin/INDOLE ACETIC ACID (Aux/IAA) repressors interact with ARFs through the shared PB1 (Phox and Bem1) domain under low-auxin conditions and thus prevent expression of auxin-responsive genes. Following the perception of auxin, Aux/IAA proteins are degraded by the SCF^TIR1/AFB^ complex to permit ARF-mediated transcription. ARF3 (also known as ETTIN), which participates in a variety of developmental processes, lacks the PB1 domain (Liu et al. 2014; Zhang et al. 2018, 2022; Su et al. 2020; McLaughlin et al. 2021). Seminal studies have revealed that ARF3 mediates auxin signaling in an Aux/IAA-independent pathway. Specifically, ARF3 directly binds auxin molecules, which dissolves interactions with process-specific transcription factors and confers auxin sensitive expression of target genes (Simonini et al. 2016, 2017; Kuhn et al. 2020). *ARF3* regulates the formation of lateral root primordia and is expressed in cells proximal to the QC, suggesting its roles in relating auxin levels to SCN specification (Root Cell Atlas, https://rootcellatlas.org/) (Marin et al. 2010; Yoon et al. 2010; Denyer et al. 2019; Wendrich et al. 2020; Kirolinko et al. 2021).

Here, we show that ARF3 constituted a complex with ARF3-INTERACTING PROTEIN1/2 (AIP1/2) and SIN3-ASSOCIATED POLYPEPTIDE OF 18 KDA (SAP18), which confined *WOX5* expression to the QC, thereby maintained the root SCNs. During SCN respecification, high auxin levels disrupted the interaction among ARF3, AIP1/2 and SAP18, which allowed the induction of *WOX5* expression and determined the new QC. Our results revealed the mechanisms underlying the regulatory roles of the ARF3-AIP1/2-SAP18 module in root SCN specification.

## Results

### *ARF3* is required for root meristem maintenance

**Figure 1:**
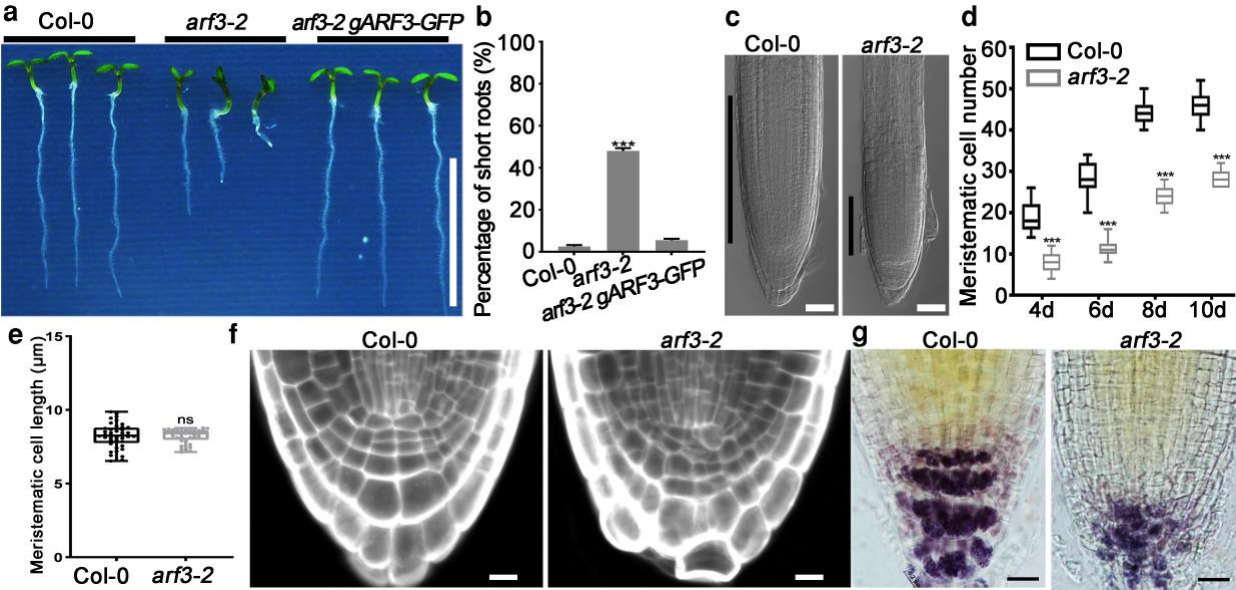
Mutations in *ARF3* disrupted maintenance of the root meristem. **a)** Root phenotype of wild-type, *arf3-2*, and *arf3-2 gARF3-GFP* seedlings at 6 d after germination (DAG). Bar = 1 cm. **b)** Percentage of short roots in **(a)**. **c)** Meristematic regions of wild-type and *arf3-2* short root at 6 DAG. Black lines indicate the length of meristem zones. Bars = 50 μm. Data are mean ± SD of 3 independent biological replicates. **d, e)** meristematic cell number **(d)** and cell length **(e)** of wild-type and *arf3-2* short root seedlings. Meristematic cell number and cell length were represented by cortex cell number and cell length in a file extending from the QC to the first expanded cell. For **(d)**, wild type at 4 DAG, n = 30; *arf3-2* at 4 DAG, n = 36; wild type at 6 DAG, n = 32; *arf3-2* at 6 DAG, n = 42; wild type at 8 DAG, n = 40; *arf3-2* at 8 DAG, n = 36; wild type at 10 DAG, n = 34; *arf3-2* at 10 DAG, n = 38. For **(e)**, wild type, n = 40; *arf3-2*, n = 40. Data are mean ± SD. Center line: median, bound of boxes: the 25th and 75th percentiles, whiskers: minimum and maximum values. **f)** Cellular arrangement was abnormal in *arf3-2* short root SCN at 3 DAG. Bars = 20 μm. **g)** Columella layers stained by I/KI solution were disrupted in the short root of *arf3-2* seedling at 5 DAG. Bars = 20 μm. ***P < 0.001 are determined by 2-tailed Student *t* test.

To assess the potential function of *ARF3* in root meristem maintenance, we analyzed the *arf3-2* mutant phenotype (Okushima et al. 2005). Compared with that of wild type, 46% of the *arf3-2* seedlings showed significantly reduced primary root length, and 42% exhibit roots shorter than 75% of wild-type length, which were referred to as the short-root phenotype (Fig 1, a and b). Three other alleles, including *ett-1*, *ett-2*, and *arf3-29*, exhibited the same phenotype (Fig. S1). The root phenotype of the *arf3-2* seedlings was complemented by introducing a *gARF3-GFP* vector (Fig 1, a and b). To determine whether the short-root phenotype was caused by meristematic defects, we examined the activity and structure of *arf3-2* root meristem. The results showed that compared with that of wild type, the meristem region was reduced in short-root *arf3-2* seedlings and meristematic cell number was significantly decreased, while meristematic cell length remained unchanged, indicating that the reduction in meristem size primarily reflects decreased cell proliferation rather than altered cell elongation (Fig 1, c–e) (Dello Ioio et al. 2007). Moreover, in the meristem of *arf3-2* short roots, QC cells exhibited abnormal shapes (Fig. 1f). The stratified structure of the columella was disturbed (Fig. 1g). However, *arf3-2* roots of normal length resembled wild type in meristem structure and meristematic cell number, except for QC shape changes in ∼49% of seedlings (Fig. S2). These results indicate that *ARF3* contributes to maintaining the root meristem.

### ARF3 specifies the root SCN by defining *WOX5* expression

**Figure 2:**
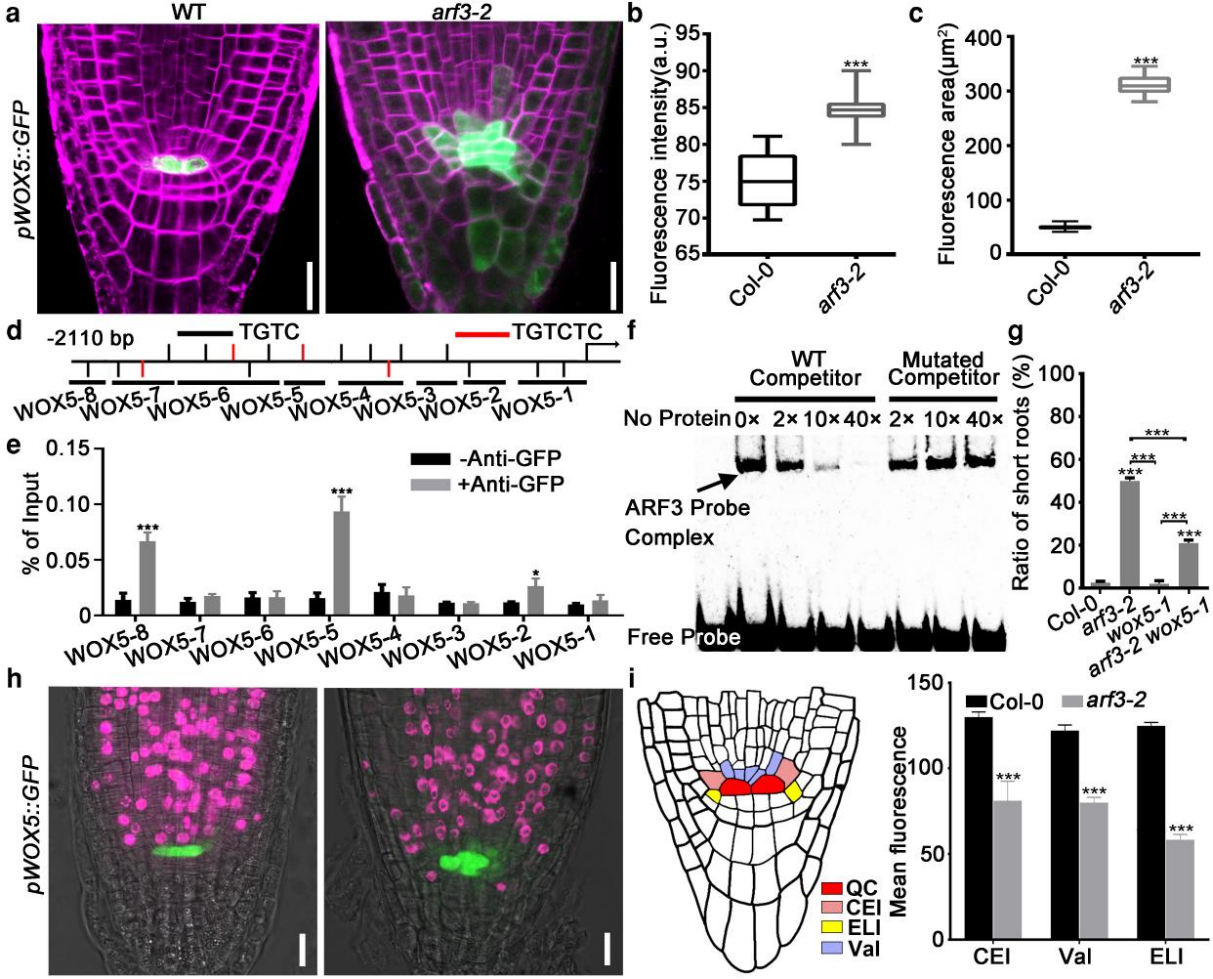
ARF3 regulated *WOX5* expression and cell division in root meristem. **a)** Expression signals of *pWOX5::GFP* were expanded into the cells lateral and proximal to QC in *arf3-2* short roots. Bars = 10 μm. **b, c)** Statistics of fluorescence intensity **(b)** and area **(c)** of *pWOX5::GFP* signals in **(a)**. Wild type, n = 30; *arf3-2*, n = 30. Data are mean ± SD. Center line: median, bound of boxes: the 25th and 75th percentiles, whiskers: minimum and maximum values. **d)** Scheme of *WOX5* promoter region. “-2110” on the left indicates 2110 bp upstream of the ATG start codon. WOX5-1 to WOX5-8 indicate the positions of promoter fragments used for ChIP-qPCR analyses. The black and red bars indicate the ARF binding elements TGTC or TGTCTC, respectively. **e)** ChIP-qPCR analyses demonstrated the association of ARF3 with the promoter regions of *WOX5*. **f)** EMSA revealed that ARF3 is directly bound to the *WOX5* promoter fragment. Oligonucleotide used for probe was derived from the promoter fragments WOX5-8 shown in **(d)**. Arrow indicates band shift. Non-labeled but not mutated oligonucleotide was able to compete for the interaction with ARF3. **g)** Percentages of the short-root phenotype in wild-type, *arf3-2*, *wox5-1* and *arf3-2 wox5-1* seedlings. **h)** EdU staining signals were obviously attenuated in the proximal and lateral stem cells of the *arf3-2* root (right) compared with those in the wild type (left). Bars = 20 μm. **i)** Quantification of EdU staining intensity in **(h)**. VaI, vascular initials; CEI, cortex/endodermal initials; ELI, epidermal/lateral root cap initials. Wild type, n = 40; *arf3-2*, n = 40. Data are mean ± SD. For **(e)** and **(g)**, data are mean ± SD of 3 independent biological replicates. For **(b)**, **(c)**, **(e)** and **(i)**, *P < 0.05 and ***P < 0.001 are determined by 2-tailed Student *t* test. For **(g)**, ***P < 0.001 are determined by ANOVA for multiple-group comparison.

To clarify the regulatory mechanisms of ARF3-mediated meristem maintenance, we conducted RNA-sequencing analysis to identify differentially expressed genes between *arf3-2* and wild-type root tips. Only *arf3-2* seedlings with the short-root phenotype, where ARF3-dependent effects are most penetrant, were included in this analysis. Among the previously determined meristem regulatory genes, the transcriptional level of *WOX5* was substantially higher in the *arf3-2* root tip than that of the wild type (Supplementary Data Set S1). Consistently, *pWOX5::GFP* signals were reinforced in the *arf3-2* root meristem and were expanded into the cells lateral and proximal to the QC (Fig 2a–c). We further examined *WOX5* expression across individual root types of different lengths using RT-qPCR. The result showed that *WOX5* transcript level was negatively correlated with root length (Fig. S3). In *arf3-2* roots of normal length, only mild expansion of *pWOX5::GFP* occurred, and only in those exhibiting changes in QC cell shape (Fig. S2). Furthermore, chromatin immunoprecipitation (ChIP)-qPCR and electrophoretic mobility shift assay (EMSA) revealed a direct association between ARF3 and *WOX5* promoter fragments, indicating that ARF3 can negatively regulate *WOX5* transcription through binding its promoter (Fig 2d–f).

Previous studies have shown that the proximally ectopic expression of *WOX5* is associated with root growth inhibition (Ji et al. 2015; Zhang et al. 2015). Thus, we tested whether the short-root phenotype of *arf3* mutant is caused by expanded *WOX5* expression. For this purpose, we crossed *arf3-2* with the *wox5-1* single mutant. Compared with that of *arf3-2*, the percentage of short roots decreased by ∼50% in the *arf3-2 wox5-1* double mutant, indicating that ARF3 regulated root growth partially by defining *WOX5* expression (Fig. 2g).

WOX5 restrains QC cell division by repressing the expression of *CYCD3;3* (Forzani et al. 2014). Therefore, we hypothesized that the ectopic *WOX5* expression in proximal and lateral stem cells resulted in suppressed cell proliferation. To test this hypothesis, we tracked cell division using 5-ethynyl-2′-deoxyuridine (EdU) staining, which detects nuclear DNA replication during the S-phase of the cell cycle (Kotogány et al. 2010). As a result, EdU staining signals were obviously attenuated in the proximal stem cells of *arf3-2* short root (Fig. 2h). Quantification of EdU staining signals demonstrated significant reduction of the intensity (Fig. 2i). However, in *arf3-2* root of normal length, EdU staining signals remained indistinguishable from wild type, indicating that cell proliferation and root elongation are preserved when *WOX5* expansion is minimal (Fig. S2). We thus focus subsequent analysis on seedlings with short-root phenotype. Together, these results indicate that ARF3 contributes to the proliferation of proximal stem cells in the root tip, at least in part by restricting *WOX5* expression into the QC.

### ARF3 forms a protein complex with AIP1/2 and SAP18

**Figure 3:**
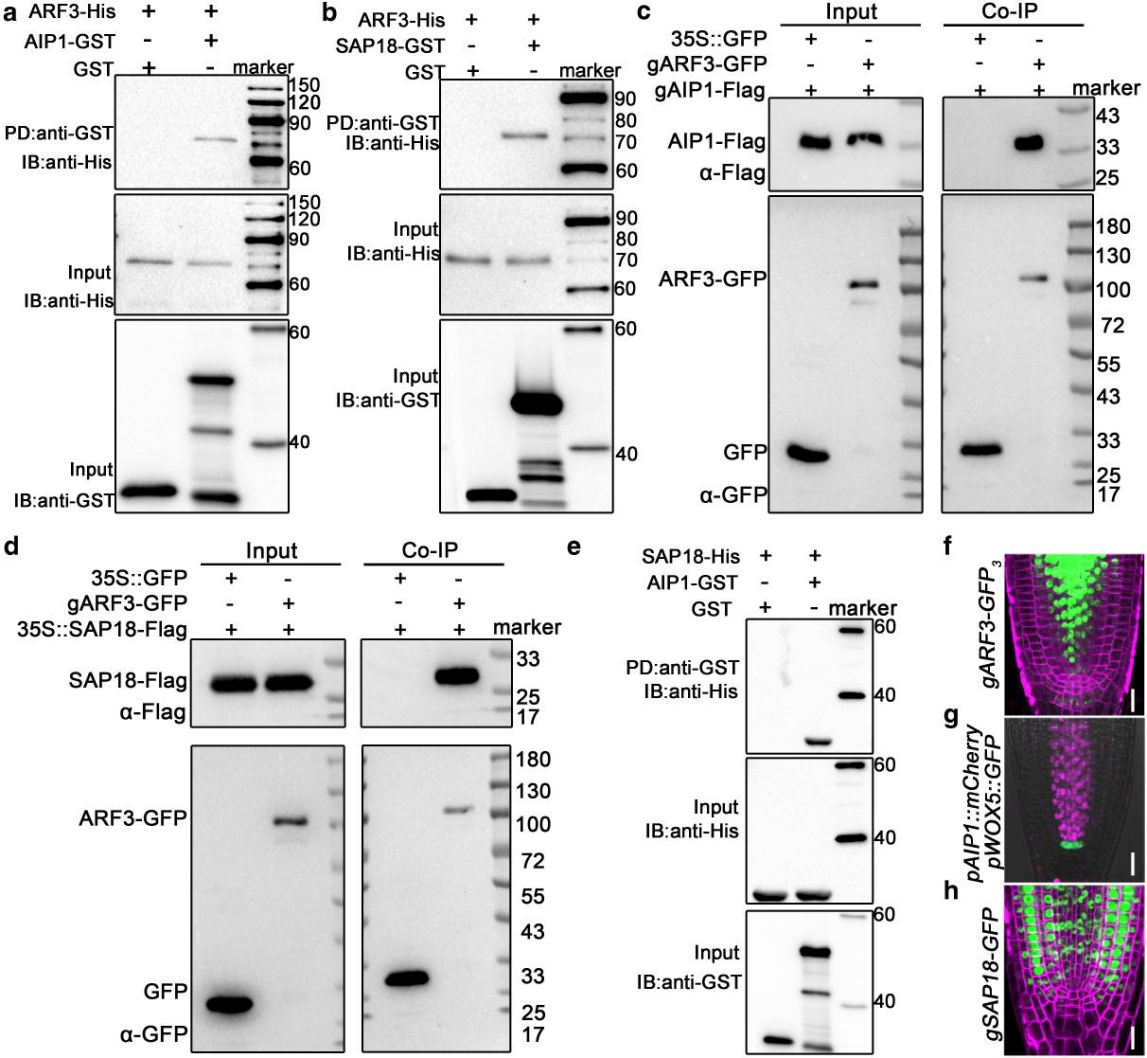
ARF3, AIP1/2, and SAP18 formed a protein complex. **a)** ARF3-His was pulled down by AIP1-GST. **b)** ARF3-His was pulled down by SAP18-GST. **c)** Co-IP assay revealed the interaction between ARF3 and AIP1. **d)** Co-IP assay indicated the interaction between ARF3 and SAP18. **e)** Pull-down assay showed the interaction between AIP1 and SAP18. **f to h)** Expression patterns of *gARF3-GFP_3_*  **(f)**, *pAIP1::mCherry pWOX5::GFP*  **(g)**, and *gSAP18-GFP*  **(h)** were visualized in root tips of complemented lines. For **(c)** and **(d)**, *gARF3-GFP gAIP1-Flag* and *gARF3-GFP 35S::AIP1-Flag* transgenic seedlings at 10 DAG were used for analysis, respectively. For **(f)**, bars = 50 μm. For **(g)**, bars = 20 μm. For **(h)**, bars = 20 μm.

To study how ARF3 regulates *WOX5* transcription, we screened for ARF3-interacting proteins by yeast 2-hybrid (Y2H) assay (Supplementary Data Set S2). As a result, we identified a leucine-rich repeats (LRRs) containing protein with unknown function, which was named ARF3 INTERACTING PROTEIN1 (AIP1) (Fig. S4; Files S1 and S2). AIP1 co-localized with DAPI in the nucleus (Fig. S5). SIN3-ASSOCIATED POLYPEPTIDE OF 18 KDA (SAP18), which is a component of the SIN3-histone deacetylase complex (HDAC), was also a potential ARF3-interacting protein (Hill et al. 2008; Qüesta et al. 2016). Interactions between ARF3 and AIP1/SAP18 were confirmed by 1-to-1 Y2H, bimolecular fluorescence complementation (BiFC), pull-down, and Co-IP assays (Fig. 3a–d; Figs. S6, S7 a and b). Pull-down and Y2H assays also revealed the interaction between AIP1 and SAP18 (Fig. 3e; Fig. S7c). Furthermore, the middle region (MR) but not the DNA-binding domain (DBD) of ARF3 could interact with AIP1 and SAP18 (Figs. S8 and S9).

Considering the detected physical interactions, we assessed whether ARF3 expression domain overlaps with that of AIP1 and SAP18 using *gARF3-GFP_3_*, *pAIP1::AIP1-mCherry*, and *gSAP18-GFP* reporter lines. As a result, co-distribution of all these 3 reporters was detected in the proximal stem cells and stele, suggesting that ARF3 assembled a complex with AIP1 and SAP18 in the cells in which *WOX5* repression occurs (Fig. 3f–h). However, the expression patterns were not completely overlapped, implying that these 3 factors may carry out independent functions in the cell types where their expression patterns diverge.

### AIP1/2 and SAP18 regulate *WOX5* expression and SCN maintenance

**Figure 4:**
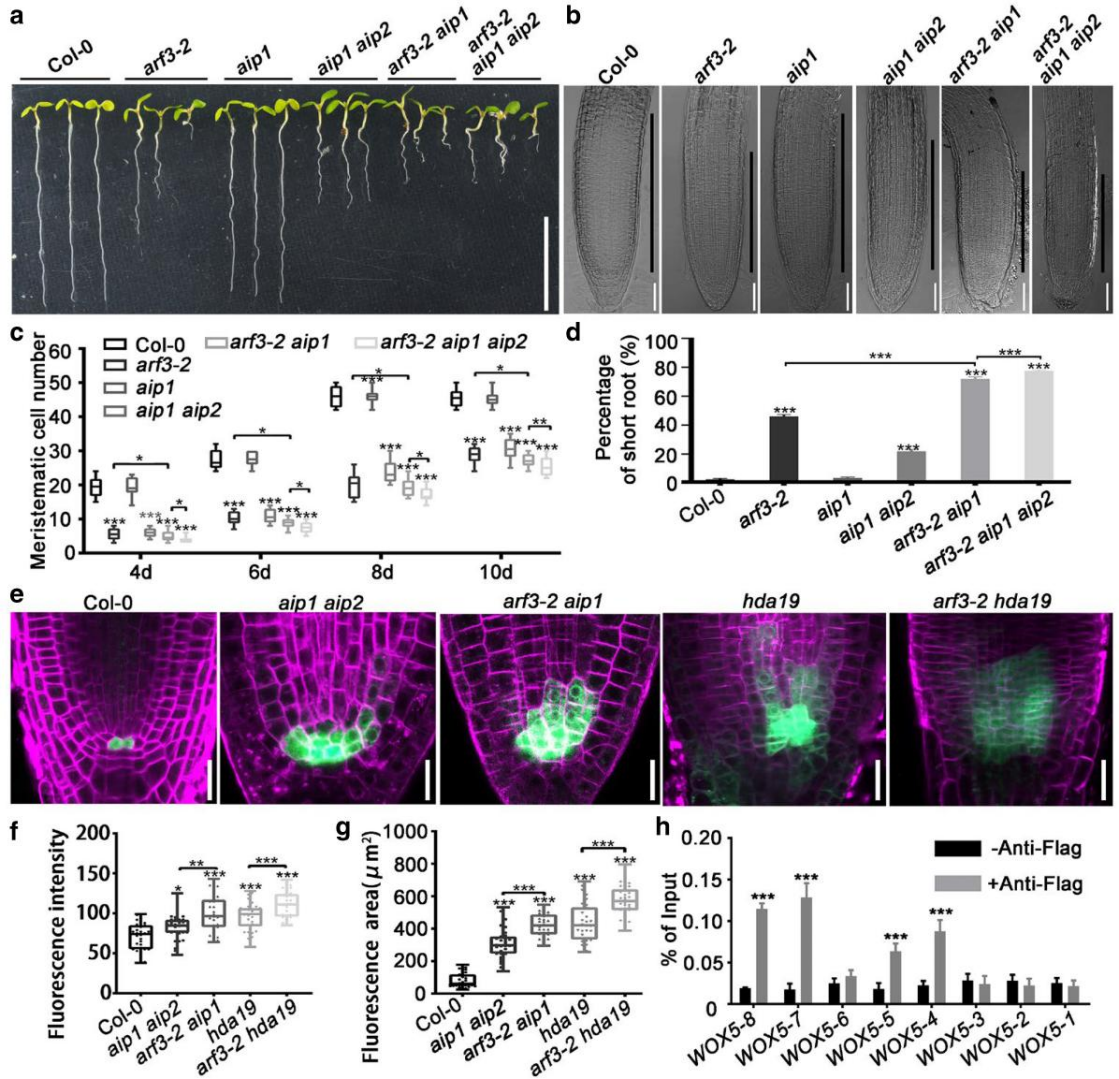
AIP1/2 and SAP18 were involved in regulating *WOX5* expression and SCN maintenance. **a)** Root phenotype of wild-type and *arf3-2*, *aip1*, *aip1 aip2*, *arf3-2 aip1*, and *arf3-2 aip1 aip2* mutant seedlings at 6 DAG. Bar = 1 cm. **b to d)** Root meristematic regions **(b)**, meristematic cell number **(c)** and percentage of short-root phenotype **(d)** of seedlings in **(a)**. For **(b)**, black lines indicate the length of meristem regions. Bars = 20 μm. For **(c)**, 4 DAG: wild type, n = 10; *arf3-2*, n = 10; *aip1*, n = 10; *aip1 aip2*, n = 10; *arf3-2 aip1*, n = 10; *arf3-2 aip1 aip2*, n = 10. 6 DAG: wild type, n = 10; *arf3-2*, n = 10; *aip1*, n = 10; *aip1 aip2*, n = 10; *arf3-2 aip1*, n = 10; *arf3-2 aip1 aip2*, n = 10. 8 DAG: wild type, n = 10; *arf3-2*, n = 10; *aip1*, n = 10; *aip1 aip2*, n = 10; *arf3-2 aip1*, n = 10; *arf3-2 aip1 aip2*, n = 10. 10 DAG: wild type, n = 10; *arf3-2*, n = 10; *aip1*, n = 10; *aip1 aip2*, n = 10; *arf3-2 aip1*, n = 10; *arf3-2 aip1 aip2*, n = 10. **e)** Expression patterns of *pWOX5::GFP* in wild-type, *aip1 aip2*, *arf3-2 aip1*, *hda19* and *arf3-2 hda19* root tips. Bars = 20 μm. **f, g)** Statistics of fluorescence intensity **(f)** and area **(g)** of *pWOX5::GFP* signals in **(e)**. Wild-type, n = 30; *aip1 aip2*, n = 30; *arf3-2 aip1*, n = 30; *hda19* n = 30; *arf3-2 hda19,* n = 30. **h)** ChIP analyses showed the association of AIP1 with the promoter regions of *WOX5*. The positions of *WOX5* promoter fragments were shown in Fig. 2d. For **(c)**, **(f)** and **(g)**, data are mean ± SD. Center line: median, bound of boxes: the 25th and 75th percentiles, whiskers: minimum and maximum values. For **(d)** and **(h)**, data are mean ± SD of 3 independent biological replicates. For **(c)**, **(d)**, **(f)**, and **(g)**, *P < 0.05, **P < 0.01, and ***P < 0.001 are determined by ANOVA for multiple-group comparison. For **(h)**, ***P < 0.001 are determined by 2-tailed Student *t* test.

We next investigated functions of AIP1/2 and SAP18 in regulating *WOX5* expression and root SCN maintenance. Through a genome-wide survey, a paralog of *AIP1* was identified and designated as *AIP2* (Fig. S10; Files S3 and S4). According to Y2H analysis, AIP2 can interact with ARF3 (Fig. S11a). Neither the *aip1* nor the *aip2* single mutant exhibited any visible phenotype (Fig. 4a–d; Fig. S11b). By contrast, 16% of the *aip1 aip2* seedlings showed short-root phenotype, indicating that AIP1 was functionally redundant with AIP2 (Fig. 4a–d). We then generated the *arf3-2 aip1* double and *arf3-2 aip1 aip2* triple mutants and examined their root phenotype. The results showed that compared with that of the *arf3-2* single mutant, root phenotype was more severe and the frequency of the short-root phenotype was significantly increased in the *arf3-2 aip1* double mutant. These phenotypes were further intensified in the *arf3-2 aip1 aip2* triple mutant (Fig. 4a–d). Moreover, in the *aip1 aip2* root meristem, *WOX5* expression signals were enhanced and expanded similar to those in *arf3-2* mutant. The reinforced and ectopic *WOX5* expression was more pronounced in the *arf3-2 aip1* double mutant and occurred in a higher proportion of roots (Fig. 4e–g).

Previous studies have shown that phenotype of the *sap18* mutant is negligible (Song et al. 2006; Larran et al. 2025). Considering SAP18 is a transcriptional repressor that recruits histone deacetylase 19 (HDA19), we examined the phenotype of the *hda19* mutant (Hill et al. 2008; Liu et al. 2009; Gu et al. 2013; Qüesta et al. 2016). The results showed that 38% of the *hda19* seedlings exhibited the short-root phenotype (Fig. S12). The *pWOX5::GFP* expression pattern in the *hda19* root meristem was similar to that of the *arf3-2* mutant (Fig. 4e–g). Moreover, the expansion of *pWOX5::GFP* signals was enhanced in the *hda19 arf3-2* double mutant compared with that in the *hda19* mutant. Therefore, AIP1/2 and SAP18 contribute to *WOX5* regulation and root SCN maintenance.

### The ARF3-AIP1/2-SAP18 module regulates histone acetylation in the *WOX5* promoter region

**Figure 5:**
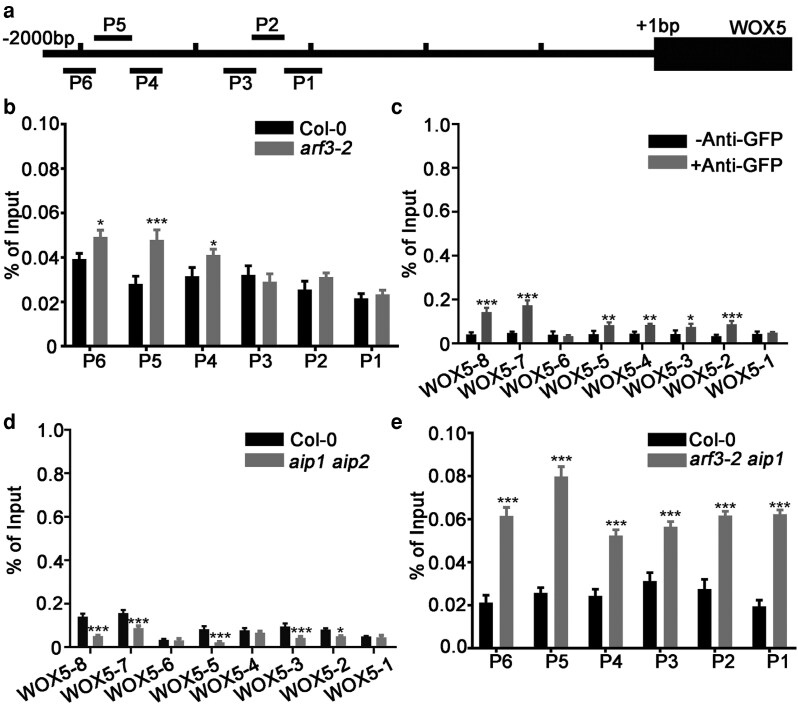
The ARF3-AIP1/2-SAP18 module mediated histone H3 acetylation. **a)** Scheme of *WOX5* promoter fragments used for the analysis of histone H3 acetylation. “-2000bp” on the left of the scheme indicates the position 2000 bp upstream of the ATG start codon. The positions of promoter fragments used for ChIP-qPCR analyses are indicated by P1 to P6. **b)** ChIP-qPCR analyses showed that compared with that of wild type, association between acetyl-histone H3 and promoter fragments of *WOX5* was increased in *arf3-2*. **c)** The association of SAP18 with *WOX5* promoter fragments were detected by ChIP-qPCR. **d)** The association of SAP18 with *WOX5* promoter fragments were reduced in the *aip1 aip2* double mutant compared with that of wild type. **e)** The association between acetyl-histone H3 and *WOX5* promoter fragments was significantly increased in the *arf3-2 aip1* double mutant compared with that of *arf3-2*. For **(c)** and **(d)**, the positions of *WOX5* promoter fragments are shown on Fig. 2d. For **(b)**, **(d)**, and **(e)**, short roots of *arf3-2*, *arf3-2 aip1*, and *aip1 aip2* mutants were used. Data are mean ± SD of 3 independent biological replicates. *P < 0.05, **P < 0.01, and ***P < 0.001 are determined by 2-tailed Student *t* test.

The abovementioned findings prompted us to test whether ARF3 acts cooperatively with AIP1/2 and SAP18 and regulates *WOX5* expression via modulating histone acetylation. We first performed ChIP-qPCR analysis using an anti-acetyl-histone H3 antibody. The results showed that compared with that of wild type, association between anti-acetyl-histone H3 and the *WOX5* promoter region increased in *arf3-2* root tip, indicating that ARF3 is required for maintaining proper histone H3 acetylation level (Fig. 5a and b). Next, we examined whether AIP1 and SAP18 can access the *WOX5* promoter similar to ARF3. According to ChIP-qPCR results, both AIP1 and SAP18 can be associated with the *WOX5* promoter region (Figs. 4h and 5c). However, direct DNA bindings were not detected by EMSAs, indicating that AIP1 and SAP18 are recruited to the *WOX5* promoter indirectly (Fig. S13). Therefore, we speculated that ARF3 directly binds to the *WOX5* promoter regions, and recruits SAP18 with the aid of AIP1/2. We then tried to verify the proposed function of AIP1/2. The results showed that in the *aip1 aip2* double mutant, the association between SAP18 and the *WOX5* promoter was significantly reduced, the interaction between ARF3 and SAP18 was abolished (Fig. 5d; Fig. S14). Mutating *AIP1* under the *arf3-2* background further increased histone H3 acetylation levels of *WOX5* promoter regions, indicating that AIP1/2 were important for SAP18 association and subsequent histone’de-acetylation of the *WOX5* promoter (Fig. 5e). The results together showed that ARF3, AIP1/2, and SAP18 formed a module to synergistically regulate *WOX5* expression through mediating histone acetylation.

We next tested whether the ARF3-AIP1/2-SAP18 module regulates *WOX5* expression interplaying with canonical auxin signaling. RT-qPCR revealed that auxin-induced transcription of *IAA12* was abolished in the *tir1 afb1 afb2 afb4 afb5* quintuple mutant (Fig. S15a). In contrast, auxin still induced *WOX5* transcription in the quintuple mutant, albeit at a reduced amplitude compared with wild type (Fig. S15b). These observations indicate that the ARF3–AIP1/2–SAP18 module can promote auxin-responsive *WOX5* expression largely independently of TIR1/AFB-based canonical auxin signaling.

### The ARF3-AIP1/2-SAP18 module regulates SCN maintenance in response to auxin dynamics

**Figure 6:**
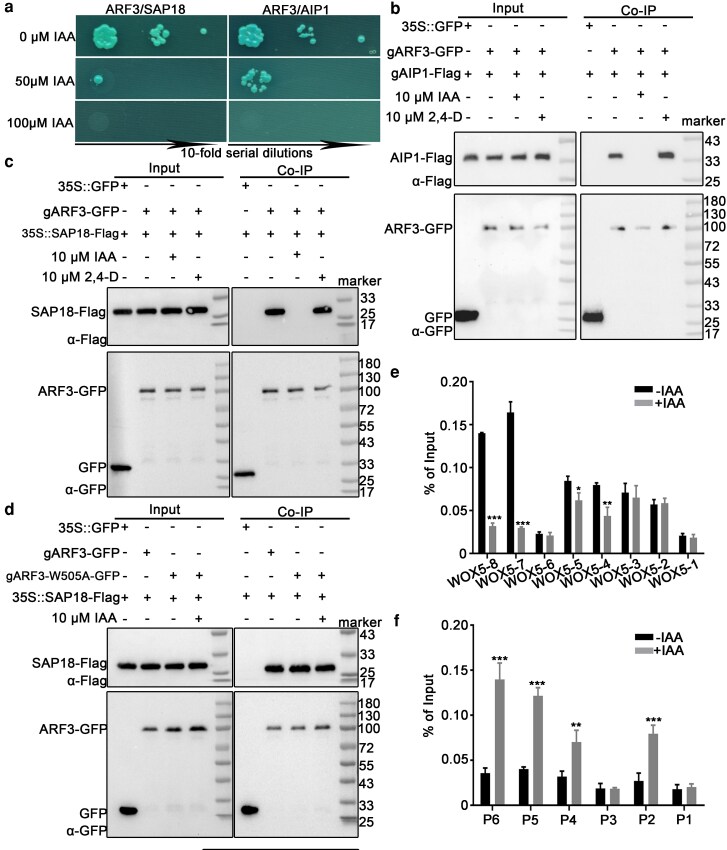
Interaction between ARF3 and AIP1/2-SAP18 was responsive to auxin levels. **a)** Y2H assays indicated that the interaction between ARF3 and AIP1/SAP18 can be disrupted by IAA treatment. **b, c)** Co-IPs showed that exogenous IAA was sufficient to eliminate the ARF3-AIP1 (b) and ARF3-SAP18 (c) interactions. **d)** Co-IP demonstrated that the interaction of ARF3(W505A)-SAP18 was insensitive to exogenous IAA. **e)** ChIP-qPCR revealed that exogenous IAA disrupted the association between SAP18 and *WOX5* promoter. The positions of *WOX5* promoter fragments are shown in Fig. 2d. **f)** ChIP-qPCR demonstrated that IAA application enhanced the association between acetyl-histone H3 and *WOX5* promoter fragments. The positions of *WOX5* promoter fragments are shown in Fig. 5a. Data are mean ± SD of 3 independent biological replicates. *P < 0.05, **P < 0.01, and ***P < 0.001 are determined by 2-tailed Student *t* test.

We subsequently examined whether the ARF3-AIP1/2-SAP18 module regulates root SCN maintenance in an auxin-dependent manner. To this end, we assessed the sensitivity of ARF3-SAP18/AIP1 interactions to exogenous IAA. The results indicated that the association between ARF3 and SAP18/AIP1 in the Y2H assays can be dissolved by adding IAA (Fig. 6a) but not by the synthetic auxin NAA and 2,4-D (Fig. S16). To provide in vivo evidence, we performed Co-IP assays using seedlings cultured in 10 μM IAA for 12 h. The results showed that treatment with IAA, but not 2,4-D, eliminated ARF3-SAP18 and ARF3-AIP1 interactions (Fig. 6b and c). A previous study has revealed that the tryptophan in position 505 (W505) of ARF3 is critical for its auxin-sensitive protein interaction (Kuhn et al. 2020). We performed Co-IP assays between a mutated version of ARF3 (W505A) and SAP18. The result demonstrated that the interaction was insensitive to exogenous IAA, indicating that the auxin-sensitive binding of SAP18 depends on W505 of ARF3 (Fig. 6d).

**Figure 7:**
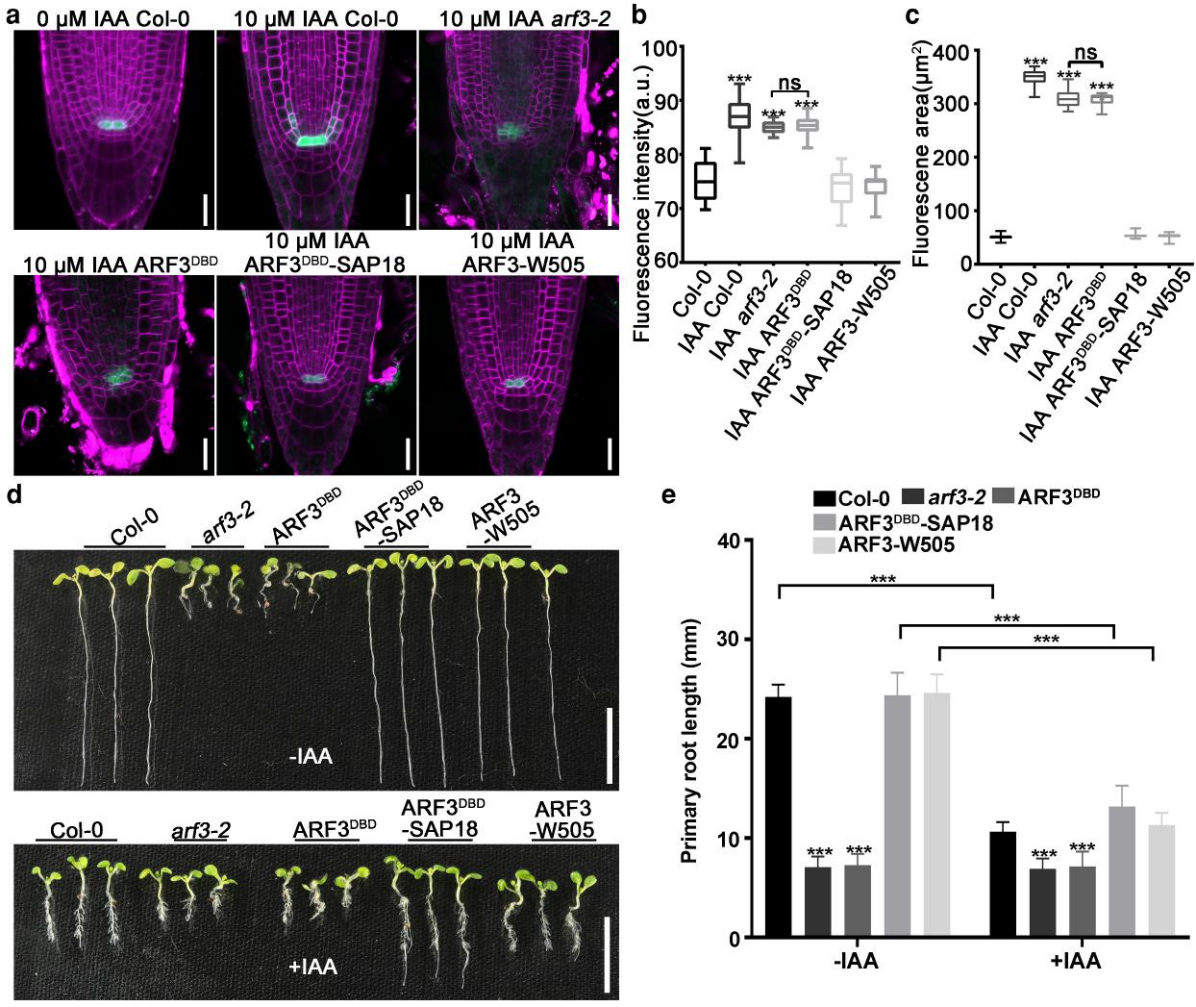
The ARF3-AIP1/2-SAP18 module regulated *WOX5* expression and root elongation in response to auxin levels. **a)** IAA application led to the expansion of *pWOX5::GFP* signals in wild type but did not alter the signal distribution in *arf3-2 pWOX5::GFP*, *arf3-2 pARF3::ARF3^DBD^ pWOX5::GFP*, *ARF3^DBD^-SAP18*, and *arf3-2 pARF3::ARF3* (*W505A*) *pWOX5::GFP* root meristem. Bars = 20 μm. **b, c)** Statistics of fluorescence intensity **(**b**)** and area **(**c**)** of *pWOX5::GFP* signals in (a). Wild type, n = 30; IAA-treated wild type, n = 30; *arf3-2 pWOX5::GFP*, n = 30; *arf3-2 pARF3::ARF3^DBD^ pWOX5::GFP*, n = 30; *ARF3^DBD^-SAP18*, n = 30; *arf3-2 pARF3::ARF3* (*W505A*) *pWOX5::GFP*, n = 30. Center line: median, bound of boxes: the 25th and 75th percentiles, whiskers: minimum and maximum values. **d)** Root phenotype of wild-type, *arf3-2 pWOX5::GFP*, *arf3-2 pARF3::ARF3^DBD^ pWOX5::GFP*, *ARF3^DBD^-SAP18* and *arf3-2 pARF3::ARF3* (*W505A*) *pWOX5::GFP* seedlings in the absence and presence of exogenous IAA. Bars = 1 cm. **e)** Statistic data of the primary root length displayed in (d). In the absence of IAA, wild-type, n = 40; *arf3-2 pWOX5::GFP*, n = 40; *arf3-2 pARF3::ARF3^DBD^ pWOX5::GFP*, n = 40; *ARF3^DBD^-SAP18*, n = 40; *arf3-2 pARF3::ARF3* (*W505A*) *pWOX5::GFP,* n = 40. In the presence of IAA, wild-type, n = 40; *arf3-2 pWOX5::GFP*, n = 40; *arf3-2 pARF3::ARF3^DBD^ pWOX5::GFP*, n = 40; *ARF3^DBD^-SAP18*, n = 40; *arf3-2 pARF3::ARF3* (*W505A*) *pWOX5::GFP,* n = 40. Data are mean ± SD. *P < 0.05, **P < 0.01, and ***P < 0.001 are determined by ANOVA for multiple-group comparison.

ChIP-qPCR was used to assess the effects of elevated auxin levels on histone H3 acetylation and *WOX5* expression. The results showed that IAA treatment suppressed the association between SAP18 and *WOX5* promoter fragments while increasing histone H3 acetylation levels in the *WOX5* promoter region (Fig. 6e and f). In accordance with previous studies, a 10-μM IAA treatment gave rise to ectopic *pWOX5::GFP* expression reminiscent of that in the *arf3-2* and *aip1 aip2* mutants (Fig. 7a–c) (Mähönen et al. 2014). In the gain-of-function *yuc1-D* mutant with elevated endogenous IAA level, the transcript level of *WOX5* was significantly higher than that of wild type (Fig. S17) (Zhao et al. 2001). Hence, increases in the auxin level can disrupt the ARF3-AIP1/2-SAP18 complex, thereby increasing *WOX5* transcription by facilitating histone H3 acetylation in its promoter region.

To explore the relevance of the ARF3-AIP1/2-SAP18 module in root development, we expressed a fusion construct containing sequences encoding the ARF3-DBD domain and the *SAP18* coding region under the *ARF3* promoter in the *arf3-2* mutant. We predicted that the ARF3^DBD^-SAP18 fusion protein cannot be dissolved by excessive auxin, enabling it to repress ectopic *WOX5* expression and alleviate the inhibition of root elongation at high auxin levels. Consistent with our prediction, under the 10-μM IAA treatment, the root growth inhibition of the *pARF3::ARF3^DBD^-SAP18 arf3-2* (*ARF3^DBD^-SAP18* for short) seedlings was attenuated compared with that of wild type (Fig. 7, d and e). Moreover, the IAA treatment did not lead to expansion of *WOX5* expression in the *ARF3^DBD^-SAP18* root meristem (Fig. 7a–c).

As controls, root elongation and *WOX5* expression in *arf3-2 pWOX5::GFP*, *arf3-2 pARF3::ARF3^DBD^ pWOX5::GFP*, *arf3-2 pARF3::ARF3* (*W505A*) *pWOX5::GFP*, and *aip1 aip2 pWOX5::GFP* seedlings were largely resistant to IAA treatment, consistent with the disruption of auxin-responsive module mediated by ARF3. Expressing only the ARF3 DNA-binding domain under the native promoter (*arf3-2 pARF3::ARF3^DBD^ pWOX5::GFP*) did not rescue the *arf3-2* root phenotype nor restore auxin responsiveness, indicating that ARF3's C-terminal protein–protein interaction region is required for functional complementation (Fig. 7; Fig. S18). These results indicated that high-level auxin inhibited root elongation at least partially by disrupting the ARF3-AIP1/2-SAP18 complex. Therefore, the ARF3-AIP1/2-SAP18 maintained root SCN by regulating *WOX5* expression in an auxin-responsive manner.

### The ARF3-AIP1/2-SAP18 module regulates SCN respecification

**Figure 8:**
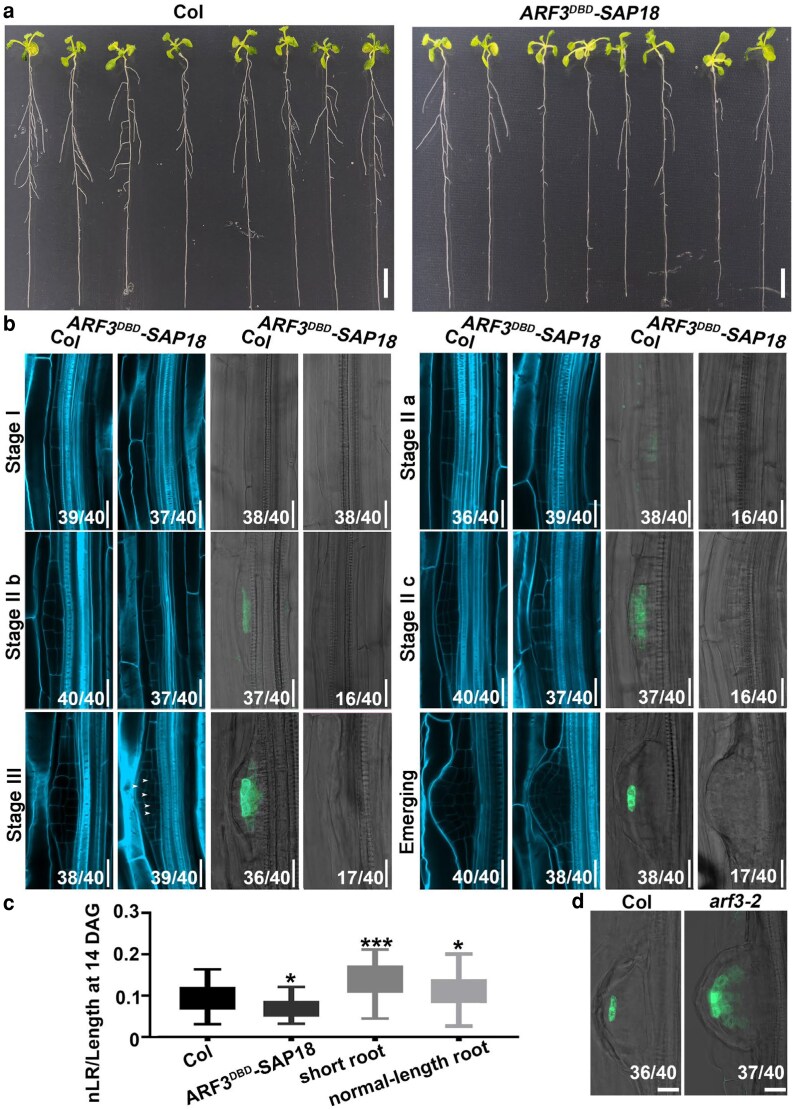
The ARF3-AIP1/2-SAP18 module regulated lateral root formation. **a)** Lateral roots formed in wild-type (left) and *ARF3^DBD^-SAP18* (right) seedlings at 10 DAG. Bars = 1 cm. **b)** Morphology and *pWOX5::GFP* expression patterns of wild-type and *ARF3^DBD^-SAP18* lateral root primordia. Stage II a-c represent 3 timepoints of stage II. Bars = 20 μm. **c)** Statistics of lateral root density of wild-type, *ARF3^DBD^-SAP18*, and *arf3-2* (short-root and normal-length root) seedlings. Wild type, n = 40; *ARF3^DBD^-SAP18*, n = 30; *arf3-2* (short root), n = 37; *arf3-2* (normal length root), n = 30. Data are mean ± SD. *P < 0.05 and **P < 0.01 are determined by ANOVA for multiple-group comparison. Center line: median, bound of boxes: the 25th and 75th percentiles, whiskers: minimum and maximum values. **d)** Expression signals of *pWOX5::GFP* in wild-type and *arf3-2* lateral root primordia at the emerging stage. Bars = 20 μm. For **(b)** and **(d)**, numbers in the bottom right corner denote frequencies of the shown phenotypes.

To examine the potential roles of the ARF3-AIP1/2-SAP18 module in de novo root SCN respecification, we first monitored lateral root formation. Compared with the wild type of control, lateral root density of the *ARF3^DBD^-SAP18* lines was significantly reduced (Fig. 8a and c). Morphological analysis demonstrated that lateral root primordia of the *ARF3^DBD^-SAP18* lines was indistinguishable from that of wild type at stage I (Fig. 8b). However, at stage II and stage III, aberrant cell division planes were observed in the *ARF3^DBD^-SAP18* primordia. Previous studies have shown that defects in *WOX5* interfere with cell division and reduce lateral root density (Tian et al. 2014; Du and Scheres 2017). We thus visualized the expression pattern of the *pWOX5::GFP* reporter. The results demonstrated that in wild type, *pWOX5::GFP* signals were first detectable at stage II became centrally localized by stage III and were ultimately confined to the QC inemerged primordia (Fig. 8b). By contrast, in 40% of the *ARF3^DBD^-SAP18* primordia, the expression signals were undetectable at stages II and III. The results showed that dissolvability of the ARF3-AIP1/2-SAP18 complex is required for *WOX5* induction and lateral root formation.

In accordance with previous studies, our results revealed an increase in the lateral root density in the *arf3-2* mutant, especially in short-root seedlings; this increase was even greater in the *arf3-2 aip1* double mutant (Fig. 8c; Fig. S19a) (Simonini et al. 2016). This suggests that disrupting the ARF3-AIP1/2-SAP18 module facilitated *WOX5* induction and consequent establishment of lateral root primordia. Consistently, overexpressing *WOX5* significantly increased the lateral root density (Fig. S20). However, the *WOX5* expression pattern was altered in the *arf3-2* mutant. In wild-type lateral root primordia at the emerging stage, *WOX5::GFP* signals were specifically localized in the QC, whereas the signals were laterally expanded in the *arf3-2* mutant (Fig. 8d). Thus, the ARF3-AIP1/2-SAP18 module functions in confining *WOX5* expression during the formation of lateral root primordia.

**Figure 9:**
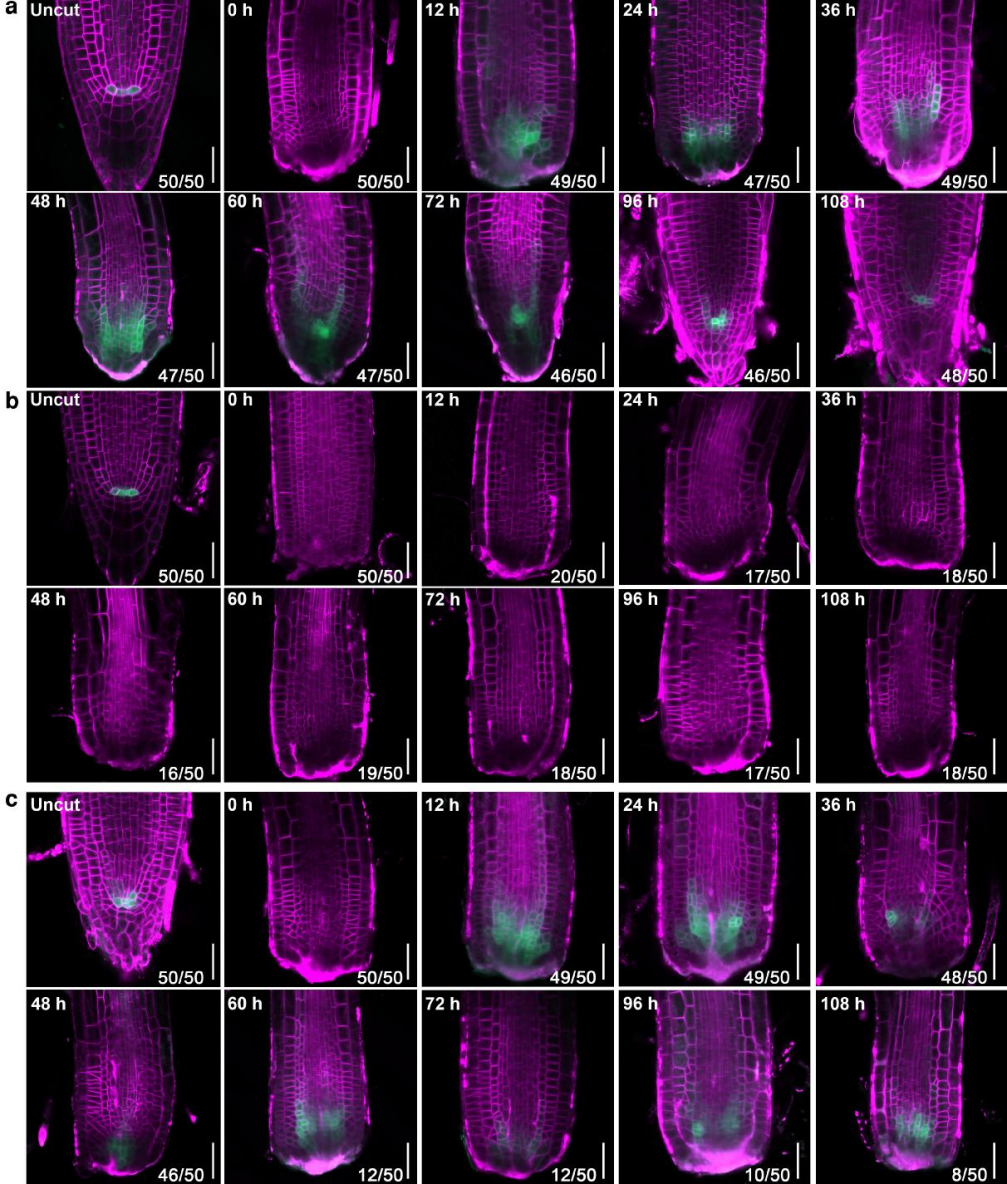
The ARF3-AIP1/2-SAP18 module participated in root tip regeneration. **a to c)** Expression pattern of *pWOX5::GFP* in root-tip regeneration procedure of wild-type **(a)**  *arf3-2*  **(b)** and *ARF3^DBD^-SAP18*  **(c)** seedlings. Bars = 20 μm. Numbers in the bottom right corner denote frequencies of the shown phenotypes.

We next investigated the involvement of the ARF3-AIP1/2-SAP18 module in root-tip regeneration. At 5 d post cut of the root tip containing the SCN, 96% of the wild-type roots accomplished regeneration. However, 36% of *ARF3^DBD^-SAP18*, 16% of *arf3-2* (short-root seedlings), and 22% of *arf3-2 aip1* roots failed to form new tips (Fig. S19b). Expressional analysis indicated that in wild-type roots, *WOX5* was ectopically expressed in endodermal and stele cells above the cut site starting at 12 h post cut (hpc) and was restricted into the new established QC at 60 hpc (Fig. 9a). However, *WOX5* expression signals were undetectable in *ARF3^DBD^-SAP18* roots with aborted regeneration, indicating that dissociation of the ARF3-AIP1/2-SAP18 complex is required for inducing *WOX5* expression (Fig. 9b). In *arf3-2* roots, the ectopic expression of *WOX5* was visible from 12 hpc but was still not confined into the QC at 96 hpc, suggesting that the ARF3-AIP1/2-SAP18 complex is responsible for confining *WOX5* expression during root-tip regeneration (Fig. 9c; Fig. S21). Consistent with their weak or negligible root phenotypes, *aip1*, *aip2*, and *sap18* single mutants did not exhibit detectable changes in lateral root density or root-tip regeneration frequency (Fig. S19). The results together showed that the ARF3-AIP1/2-SAP18 module acted as an auxin-responsive switch for *WOX5* expression and thus regulated root SCN respecification.

## Discussion

The graded distribution of auxin instructs root SCN; however, the underlying regulatory mechanism remains to be explored (Overvoorde et al. 2010; Roychoudhry and Kepinski 2022). In this study, we determined that the ARF3-AIP1/2-SAP18 module regulated both maintenance and respecification of the root SCN in response to auxin dynamics.

**Figure 10:**
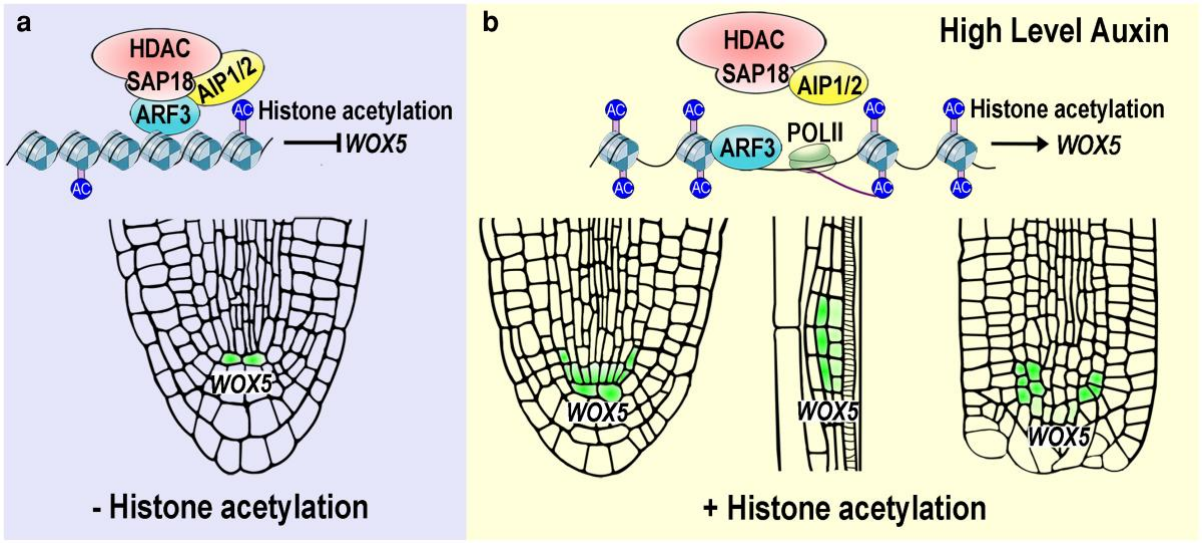
A model for regulatory roles of the ARF3-AIP1/2-SAP18 module in maintenance and respecification of root SCN. **a)** In the cells proximal to the QC, ARF3 directly binds the *WOX5* promoter, and recruits HDAC synergistically with AIP1/2. As a result, reduced histone acetylation leads to chromatin condensation and repression of *WOX5* transcription. **b)** Upon excessive auxin in root tips during formation of lateral root primordia or root-tip regeneration, the ARF3-AIP1/2-SAP18 module are dissolved, which leads to an increase in histone acetylation and allows *WOX5* expression. Green areas indicate the overlapping regions of ARF3, AIP1/2 and SAP18. Red spots represent the expressing domains of *WOX5*.

ARF3, AIP1/2 and SAP18 were coexpressed in cells proximal to the QC and formed a complex by direct interactions. The ARF3-AIP1/2-SAP18 complex bound to the *WOX5* promoter, decreased H3 histone acetylation by recruiting the HDAC, and thus restricted *WOX5* expression to the QC. Disrupting the ARF3-AIP1/2-SAP18 complex by mutation or excessive auxin led to proximal and lateral expansion of *WOX5* expression, which in turn inhibited root elongation via repressing cell division (Fig. 10a). During lateral root development, poplar transport gave rises to auxin accumulation in the central cells of the primordia (Benková et al. 2003; Ditengou et al. 2008). In the root-tip regeneration process, local auxin biosynthesis is induced in the vicinity of the cut site (Matosevich et al. 2020). The elevated auxin level dissolved the ARF3-AIP1/2-SAP18 complex, which resulted in ectopic *WOX5* expression in these regions (Fig. 10b). Subsequently, as auxin gradually concentrated in the progenitor cells, the reformed ARF3-AIP1/2-SAP18 complex progressively confined *WOX5* expression to the newly established QC. Therefore, the ARF3-AIP1/2-SAP18 module acted as an auxin-responsive switch. In the region proximal to the QC, where the auxin level is lower than that in the QC, this switch is turned off to ensure proper expression pattern of *WOX5* and thus maintains the SCN. During lateral root formation or root-tip regeneration, this switch is turned on by the accumulated auxin to allow *WOX5* expression and is then gradually turned off in areas except for re-established QC, resulting in SCN respecification.

The *arf3-2* mutant displays a partially penetrant short-root phenotype (46% of seedlings), suggesting that ARF3 function may be buffered in this context. Incomplete penetrance has been documented in other auxin signaling mutants, such as *tir1*/*afb* higher-order combinations, where residual activity from paralogous components can sustain partial function (Dharmasiri et al. 2005). *ARF3* has previously been shown to act redundantly with its paralog *ARF4* in multiple developmental processes, including organ asymmetry, heteroblasty, and lateral root growth (Pekker et al. 2005; Fahlgren et al. 2006; Hunter et al. 2006; Marin et al. 2010; Iwasaki et al. 2013). It is therefore plausible that *ARF4* contributes to root SCN specification when *ARF3* is lost. Additionally, we cannot exclude the possibility that other transcription factors, potentially including non-ARF factors that interact with AIP1/2-SAP18, may partially compensate for ARF3 loss. Future work will be needed to systematically test these compensatory pathways.

A recent study has shown that PLT and SCR expression signals overlap in the SCN and create a maximal synergy in the QC. The PLT-TCP-SCR complex induces *WOX5* expression during embryogenesis, root SCN maintenance, lateral root formation, and root-tip regeneration (Shimotohno et al. 2018). We propose that the dissociation of the ARF3-AIP1/2-SAP18 complex generated a permissive environment for *WOX5* expression, wherein the PLT-TCP-SCR complex initiated the transcription. Accordingly, excessive auxin led to the ectopic expression of *WOX5* in cells lateral and proximal to the QC, where PLT, TCP, and SCR are expressed at high levels (Fig. 6f) (Mähönen et al. 2014).

Based on the results of transient protoplast assays, ARF family transcription factors can be divided into 2 groups: transcriptional activators and repressors (Ulmasov et al. 1999). It has been well elucidated that activator ARFs regulate transcription by interacting with auxin-sensitive Auxin/INDOLE-3-ACETIC-ACID repressors (Dharmasiri et al. 2005; Szemenyei et al. 2008; Wu et al. 2015). Recent studies on ARF3 have begun to elucidate the mechanisms underlying the transcription mediated by repressor ARFs (Simonini et al. 2016, 2017; Kuhn et al. 2020). The results demonstrated that ARF3/ETTIN directly binds auxin and, under low-auxin conditions, forms an ARF3-TPL/TPR-HDA19 complex that represses target genes in reproductive tissues via H3 deacetylation. Our work identifies a distinct, root-specific configuration in which ARF3 associates with AIP1/2 and SAP18, and this ARF3-AIP1/2-SAP18 complex recruits HDA19 to the *WOX5* promoter to control histone H3 acetylation and *WOX5* expression.

SAP18 is a well-established component of plant SIN3-HDAC complexes, and previous studies have demonstrated physical interaction between SAP18 and HDA19 as well as HDAC-dependent transcriptional repression (Song et al. 2006; Hill et al. 2008; Liu et al. 2009; Gu et al. 2013; Qüesta et al. 2016). However, the SAP18-HDA19 interaction was not directly examined in the context of *WOX5* regulation in this study. Our results show that mutation of *HDA19* leads to the short-root phenotype and an expansion of *pWOX5::GFP* signals (Fig. 4, e–g; Fig. S11). In the *hda19 arf3-2* double mutant, the expansion of *pWOX5::GFP* signals is further enhanced compared with that in the *hda19* mutant. Together, these observations support a role for HDA19 in *WOX5* regulation, potentially acting in association with the ARF3-AIP1/2-SAP18 complex.

TPL/TPR and SAP18 are EAR-motif binding co-repressors that recruit HDA19 to target loci and thereby suppress downstream transcription (Kagale and Rozwadowski 2011). Several transcription factors, including multiple EAR-containing regulators, interact with both TPL/TPR and SAP18, indicating that these cofactors can act in partially overlapping chromatin-remodeling pathways (Kagale et al. 2010; Kagale and Rozwadowski 2011; Causier et al. 2012; Baile et al. 2021). Consistent with this view, TPL/TPR complexes have been implicated in maintenance and regeneration of the root meristem (Pi et al. 2015; Ana Espinosa-Ruiz et al. 2017; Shen et al. 2025). The auxin-dependent loss of ARF3-AIP1/2-SAP18 interaction shown in Fig. 6b and c could coexist with, and potentially be complemented by, auxin-triggered disruption of ARF3-TPL/TPR complexes. Thus, ARF3 can connect to HDA19 via at least 2 alternative corepressor assemblies, ARF3-TPL/TPR-HDA19 and ARF3-AIP1/2-SAP18-HDA19, and our genetic, biochemical, and chromatin data support a predominant role of the latter pathway at *WOX5* in the root SCN. Genetic interactions of *HDA19* with *SAP18* and *AIP1/2* will strengthen this conclusion and deserve future investigation.

We surveyed *AIP1* and *AIP2* in available genome sequences of 40 plant species, representing algae, mosses, ferns, gymnosperms, and angiosperms. Orthologs were identified in all the main lineages, except for algae, suggesting that *AIP1/2* originated in the common ancestor of land plants (Figs. S4 and S10). Although proto-ARF has been identified in algae, it does not mediate auxin-dependent transcription (Mutte et al. 2018; Martin-Arevalillo et al. 2019). Thus AIP1/2 may have coevolved with ARFs in plants after the water-to-land transition and were gradually incorporated into the auxin response. Moreover, AIP1 and SAP18 physically interact with several repressor-ARFs besides ARF3 (Fig. S22). Potential involvement of the ARF-AIP1/2-SAP18 in transcriptional regulation mediated by repressor ARFs is worthy of further study.

Expressing the *ARF3^DBD^-SAP18* fusion under the *ARF3* promoter fully rescued the short-root phenotype of *arf3-2* (Fig. 7d and e). However, upon IAA treatment, *ARF3^DBD^-SAP18* seedlings still displayed reduced root elongation relative to untreated controls, although the inhibition was less severe than in wild type. Lateral root formation was decreased but not abolished, and 36% of *ARF3^DBD^-SAP18* roots failed to regenerate new tips. These findings indicate that ARF3^DBD^-SAP18 can efficiently repress *WOX5* and maintain SCN activity under relatively low auxin concentrations, whereas high auxin levels in lateral root primordia, regenerating root tips, or under exogenous IAA may partially compromise its function, leading to incomplete rescue.

## Methods

### Plant materials and growth conditions

Arabidopsis (*Arabidopsis thaliana*) ecotype Col-0 was used as wild type background in this study. Mutants were generated under the Col-0 background, except for *ett-1* (CS8554) and *ett-2* (CS8555), which were generated under the Ws-2 background, and *arf3-29* (Liu et al. 2014) under the Ler background. The *arf3-2* (CS24604) mutant was obtained from the Arabidopsis Biological Resource Center. The *tir1 afb1 afb2 afb4 afb5* quintuple mutant was kindly provided by Dr. Mark Estelle (University of California at San Diego). The *arf3-2 aip1* double mutants were obtained by crossing the *arf3-2* and *aip1* single mutants. The *aip2* mutant was generated using CRISPR/Cas9 technology. The *aip1 aip2* double mutant was obtained by crossing *aip2* with *aip1*. The *ARF3^DBD^-SAP18* lines were obtained by crossing the *pARF3::ARF3^DBD^-SAP18* seedlings with *arf3-2*. The *ARF3^DBD^-SAP18 pWOX5::GFP* line was generated by crossing the *ARF3^DBD^-SAP18* with *pWOX5::GFP* seedling. Seeds were surface-sterilized and then cultured on half-strength Murashige and Skoog (MS) medium (pH 5.7) for 3 d at 4 °C. They were then transferred to sterile conditions at 22 °C with a 16-h-light:8-h-dark photoperiod (spectrum: 400 to 700 nm; illumination intensity: 80 to 90 μmol m^−2^s^−1^).

### Plasmid construction and plant transformation

A genomic fragment of 5,226 bp containing a 2,056-bp sequence upstream of the ATG start codon and the coding region without the stop codon of *ARF3* was amplified by PCR and cloned into the pGK-GFP3 vector (kindly provided by Dolf Weijers, Wageningen University) to generate the *gARF3-GFP_3_* expression vector and was cloned into PMDC107 (CD3-748, Gateway) to generate the *gARF3-GFP* vector. A genomic fragment of 4,201 bp containing a 2,054-bp region upstream of the ATG start codon and the coding sequence without the stop codon of *AIP1* (AT5G21090) was amplified by PCR and cloned into pEarlyGate302 (Gateway) to generate the *gAIP1-Flag* vector. So, *35S::AIP1-GFP* construct was obtained by recombining the *AIP1* coding region into the PMDC43 vector (CD3-741, Gateway). A genomic 4,469-bp fragment containing a 3,129-bp sequence upstream of the ATG start codon and the coding region without the stop codon of *SAP18* was amplified by PCR and cloned into PMDC107 (CD3-748, Gateway) to generate the *gSAP18-GFP* vector. *35S::SAP18-Flag* construct was obtained by recombining the coding region of *SAP18* into the pEarlyGate202 vector (Gateway). The *pAIP1::AIP1-mCherry* construct was generated by cloning a 2,054-bp sequence upstream of the ATG start codon and the coding sequence without the stop codon of *AIP1* into the 2300-mCherry vector. The *pARF3::ARF3^DBD^-SAP18* vector was constructed by cloning a 2,056-bp sequence upstream of the ATG start codon, of *ARF3*, the coding region of DBD domain of *ARF3* and the coding region without the stop codon of *SAP18* into Prok-eGFP (ABRC, CD3445). To construct the *gARF3(W505A)-GFP* vector, a 2,056-bp sequence upstream of start codon and the coding sequence of *ARF3*, with the tryptophan (W) to alanine (A) substitution at position 505, were cloned into the PZP211-GFP vector. The *pARF3::ARF3^DBD^* vector was generated by cloning a 2,056-bp sequence upstream of the ATG start codon and the coding region of DBD domain of *ARF3* into Prok-eGFP (ABRC, CD3445). Vectors were transformed into wild-type Arabidopsis (Col-0) and *arf3-2*, *aip1* or *sap18* (SALK_023663C) mutant seedlings, respectively, using the floral dip method. All the primer sequences are listed in Supplementary Data Set S3.

The *aip1* and *hda19* mutants were generated using CRISPR/Cas9-mediated genome editing as previously described (Feng et al. 2013). Specifically, 2 high-specificity target sequences in the *AIP1* (5′-GAGTTGGATCCCAGCTCTGG-3′) and the *HDA19* (5′-GGCTCTGTCAAGCTTAACCACGG-3′) coding sequences were selected, respectively. These sequences were independently cloned into the CRISPR/Cas9 binary vector BGK03 (Biogle, Hangzhou, China). Transgenic seedlings were generated using the floral dip method. Primary transformants (T1 generation) were initially selected based on hygromycin resistance. Genomic DNA was extracted from resistant seedlings and subjected to PCR amplification of *AIP1* and *HDA19* locus, followed by direct sequencing to verify editing events. In the subsequent T2 generation, seedlings showing both hygromycin sensitivity and inherited mutations at the target site were selected for further analysis.

### Evolutionary and phylogenetic analysis

The 1000 Plant Transcriptome database (http://www.onekp.com) was used to screen for homolog sequences of AIP1 and AIP2 (Matasci et al. 2014). Phylogenetic trees of the AIP1/2 sequences were generated using the maximum-likelihood method with bootstrap values from 1000 replicates by MEGA-X and annotated with the Interactive Tree of Life resource (https://itol.embl.de/) according to previous studies (Zhao et al. 2019). LRR repeats of the Arabidopsis AIP1 and AIP2 were predicted using the Conserved Domain Database of NCBI (Marchler-Bauer et al. 2017). MEME software was used to predict the conserved motifs in the AIP1/2 homologs (http://meme.nbcr.net/meme/cgi-bin/meme.cgi).

### Confocal microscopy

Samples for confocal microscopy analysis were prepared as previously described (Meng et al. 2017). Root plasma membrane was stained with FM4-64 (Invitrogen, F34653) to facilitate structural observation using a Zeiss LSM 980 Airyscan 2 confocal microscope. Files were assembled in ZEN (Zeiss system). For the morphological analysis of lateral root primordia, seedlings at 10 DAG were fixed in 4% paraformaldehyde (w/v, in PBS) under low vacuum conditions (37 mbar, equivalent to 690 mmHg) for 30 min at room temperature, washed twice with 1× PBS, and then cleared in Clearsee solution (10% xylitol/15% sodium deoxycholate/25% urea) for 24 h. Cleared specimens were subsequently stained with Calcofluor White Stain (Sigma-18909) for 12 h, rinsed in Clearsee (≤30 min) (Ursache et al. 2018), and examined using the confocal microscope.

### Y2H assay

Y2H analyses were performed according to the manufacturer's instructions (Clontech). The *ARF3* coding sequence was cloned into the pGBKT7 vector, while the *AIP1* and *SAP18* coding sequences were cloned into pGADT7 vector. Y2H assays were performed using a cDNA library constructed using Arabidopsis seedling. Transformants were plated on SD/-Leu/-Trp medium and SD/-Ade/-His/-Leu/-Trp/+AbA/+X-α-gal selection medium.

### BiFC assay

*ARF3*, *AIP1* and *SAP18* coding sequences were cloned into the pENTR/D-TOPO vector (Invitrogen, K240020SP) and were fused by LR reaction to the N terminus or C terminus of YFP, respectively. Vectors were then transformed into *Agrobacterium* GV3101 and injected into *Nicotiana benthamiana* leaves. Fluorescence signals in the epidermal cells were examined using confocal microscope 3 d after injection. To assess the interation between ARF3 and SAP18 in the *aip1 aip2* double mutant, protoplasts were isolated from 7DAG seedlings according to protocols described previously (Yoo et al. 2007). Briefly, seedlings were sliced and incubated in an enzyme solution (1.5% cellulase R10 and 0.4% macerozyme R10) for 4 h. Then vectors were transformed into protoplasts using PEG-mediated transformation method. After 12 to 16 h incubation, Fluorescence signals in the protoplasts were examined using confocal microscopy.

### Pull-down assay

*AIP1* and *SAP18* coding sequences were cloned into pGEX-4T-1 vector, while *ARF3* coding sequence was cloned into the pET28a vector. Vectors were transformed into *E. coli* Transetta DE3 cells (TransGen Biotech) to produce GST- or His-fused proteins. Glutathione Sepharose beads (GE Healthcare) and GST-tagged protein were incubated together at 4 °C for 4 h. After adding His-tagged proteins, beads were incubated at 4 °C for 4 h. The beads were then washed 3 times in wash buffer (500 mM NaCl, 2.7 mM KCl, 10 mM Na_2_HPO_4_, 1.8 mM KH_2_PO_4_, 1 mM PMSF, and 0.05% NP-40), then boiled in loading buffer at 99 °C for 10 min. Western blot was performed using anti-GST (TransGen, Cat# HT601) or anti-His (TransGen, Cat# HT501) antibody, respectively.

### Co-IP assay

Seedlings were harvested and ground in liquid nitrogen and then transferred into lysis buffer (50 mM Tris-HCl, 150 mM NaCl, 1 mM EDTA, 0.2% Triton X-100, 1 mM DTT, 1 mM PMSF, 10% glycerol, 10 mM NaF, 20 mM Na_3_VO_4_, and inhibitor cocktail). After centrifugation, the supernatant was incubated with GFP-Trap (Chromotek, gtma-20) overnight at 4 °C. After washing 3 times suing wash buffer (50 mM Tris-HCl, 150 mM NaCl, 1 mM DTT, 1 mM PMSF, 0.05% Triton X-100, and protease inhibitor cocktail), beads were resuspended in loading buffer and boiled at 99 °C for 10 min. Western blot was performed with anti-Flag or anti-GFP antibody, respectively.

### RNA-sequencing

Root tips of wild-type and *arf3-2* seedlings (short-root phenotype) were collected at 5 DAG for RNA-sequencing. Clean reads were mapped to the TAIR10 genome. Genes with expression-level fold-changes > 2 and false discovery rate < 0.05 were selected as differentially expressed genes (Data Set S1).

### RT-qPCR

Total RNA was extracted from 100 mg of seedling material using an Ultrapure RNA kit (CWBIO), after which cDNA was synthesized from 2 μg of total RNA using the FastKing RT Kit (TIANGEN) according to the manufacturer's protocol. qRT-PCR analyses were performed on the LightCycler 96 Instrument (Roche) with the SYBR Green SuperReal PreMix Plus (TIANGEN). All individual reactions were performed with 3 biological replicates. The primers used for qRT-PCR analysis were listed in Data Set S3.

### ChIP-qPCR analysis

ChIP assays were performed as previously described (Meng et al. 2017). Firstly, 2 g of seedlings collected at 10 DAG were treated with’1% (v/v) formaldehyde in GB buffer (0.4 M sucrose, 10 mM Tris-HCl, 1 mM EDTA) for cross-linking. After the vacuum infiltration for 20 min at room temperature, 125 mM glycine was added to quench the cross-linking solution. The chromatin suspension was sheared by sonication to generate DNA fragments (0.25 to 1 kb). Immunoprecipitations were performed with anti-GFP (Roche, 11814460001), anti-Flag (Abmart, M20008S) or anti-acetyl-Histone H3 (Active motif, 39139) antibodies. The immune-precipitated DNA fragments were then examined by qPCR.

### EMSA

EMSAs were performed using a LightShift Chemiluminescent EMSA Kit (Thermo, 20148) following the manufacturer's protocol as previously described (Cheng et al. 2013). To generate the mutated competitors, 4 bases in the auxin-responsive element were altered (TGTC to ACAG).

### I/KI staining

Seedlings collected at 7 DAG were stained for 10 s in I-KI solution and then mounted on slides with HCG solution (chloroacetaldehyde:water:glycerol = 8:3:1, w/v/v). Samples were immediately examined using an Olympus BX-51 microscope and photographed.

### Auxin treatments

Seeds from *arf3-2 pWOX5::GFP*, *arf3-2 pARF3::ARF3^DBD^ pWOX5::GFP*, *ARF3^DBD^-SAP18*, and *arf3-2 pARF3::ARF3* (*W505A*) *pWOX5::GFP* lines were cultured on half-strength MS medium (pH 5.7). At 4 DAG, seedlings were treated with 10 µM IAA for 12 h. Before being examined using a confocal microscopy, root tips were stained with FM4-64. For root phenotypic analysis, 4-DAG seedlings were treated with 10 µM IAA for 4 d and used for further analysis.

### EdU staining

EdU staining was performed using the Click-iT EdU Alexa Fluor 555 Imaging kit (Invitrogen, C10638) according to the provided instructions. Seedlings collected at 5 DAG were incubated with 2.5 μM EdU for 4 h and then examined using a LSM 980 Airyscan 2 confocal microscopy (Carl Zeiss).

### Accession numbers

Sequence data in this article can be found in the Arabidopsis Genome initiative or GenBank/EMBL databases under the following accession numbers: *ARF3* (At2g33860), *SAP18* (At2g45640), *AIP1* (At5g21090), *AIP2* (At3g43740), *HDA19* (At4g38130), *WOX5* (AT3G11260), *ACTIN2* (At3g18780), *YUC1*(At4g32540), and *IAA12*(At1g04550).

## Supplementary Material

koag108_Supplementary_Data

## Data Availability

The raw RNA-sequencing data generated in this study was submitted to NCBI under the BioProject accession number: PRJNA1420234.
