# Supplementary material for: The Arabidopsis ARF3-AIP1/2-SAP18 module specifies the root stem cell niche in response to auxin
Source: Plant Cell. 2026 Apr 10;38(5):koag108. doi: 10.1093/plcell/koag108 (PMC13221652; doi:10.1093/plcell/koag108)
Supplement: koag108_Supplementary_Data [file koag108_supplementary_data.zip › Revised Supplementary Figures.pdf]

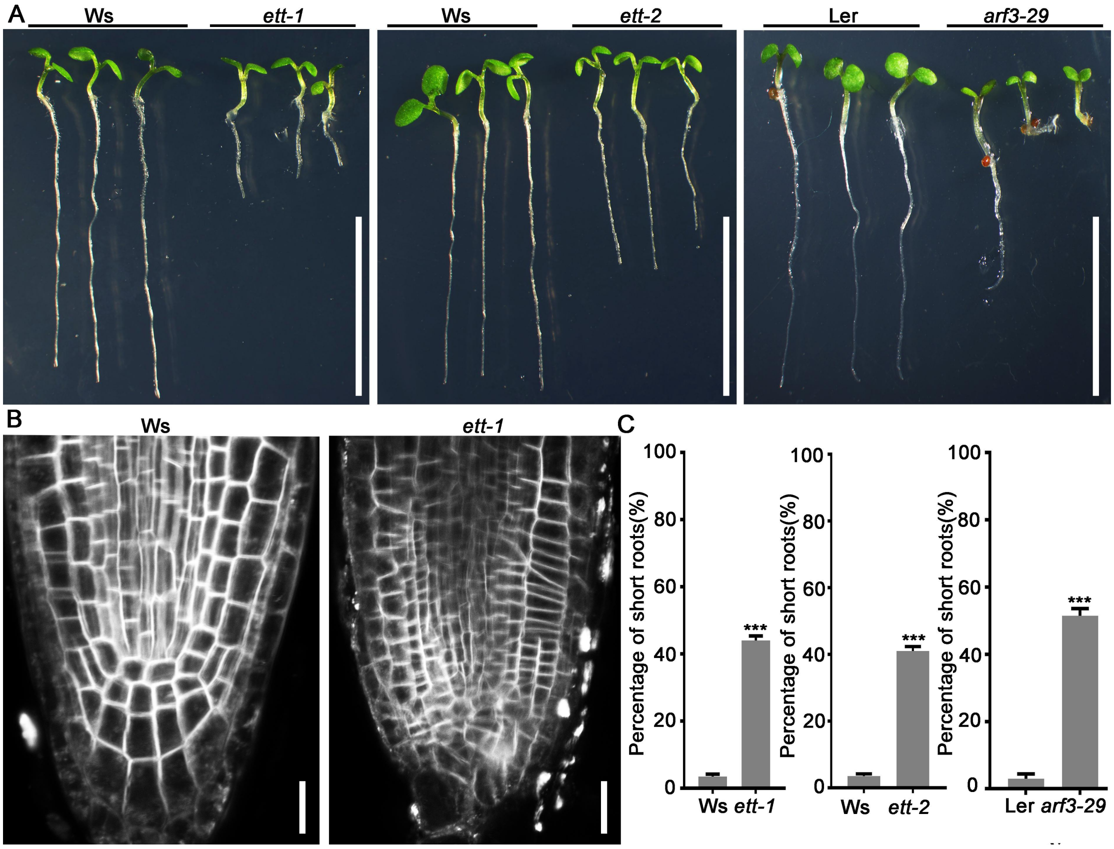

Supplementary Figure S1. Root Phenotype of different *arf3* mutant alleles. (Supports Figure 1) (A) Short-root phenotypes of the *ett-1*, *ett-2* and *arf3-29* seedlings at 5 DAG. Bars = 1 cm. **(B)** Abnormal cellular arrangement in *ett-1* root SCNs at 5 DAG. Bars = 20 μm. **(C)** Percentages of *ett-1*, *ett-2* and *arf3-29* seedlings with short-root phenotype at 5 DAG. Data are mean ± s.d. of three independent biological replicates. ****P* < 0.001 is determined by two-tailed Student's *t*-test.

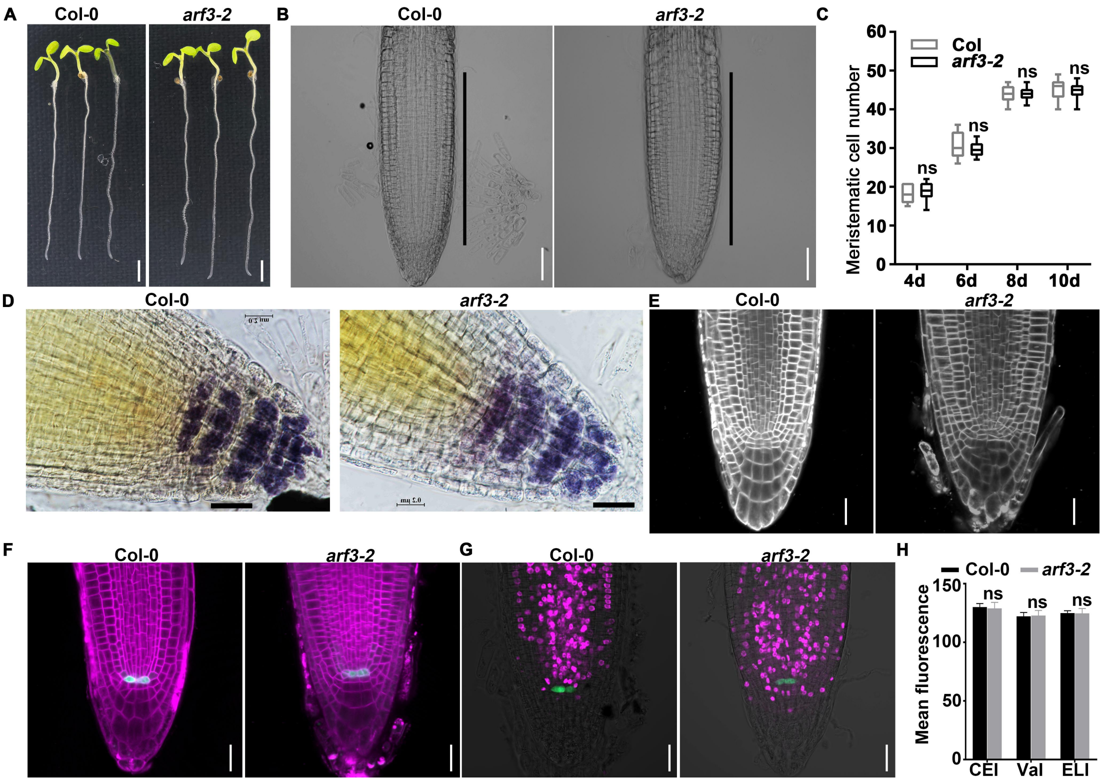

Supplementary Figure S2. Phenotypic analysis of *arf3-2* seedlings with normal-root length. (Supports Figures 1) (A) Root phenotype of wild-type and *arf3-2* seedlings at 6 days DAG. Bars = 2 mm. **(B)** Meristematic regions of wild-type and *arf3-2* seedlings at 6 DAG. Black lines indicate the length of meristem zones. Bars = 50 μm. **(C)** Number of meristematic cells of wild-type and *arf3-2* seedlings. Number of meristematic cells were represented by cortex cell number in a file extending from the QC to the first expanded cell. Wild type at 4 DAG, n = 10; *arf3-2* at 4 DAG, n = 10; wild type at 6 DAG, n = 10; *arf3-2* at 6 DAG, n = 10; wild type at 8 DAG, n = 10; *arf3-2* at 8 DAG, n = 10; wild type at 10 DAG, n = 10; *arf3-2* at 10 DAG, n = 10. Data are mean ± s.d. Statistical significance was determined by ANOVA for multiple-group comparison. Center line: median, bound of boxes: the 25th and 75th percentiles, whiskers: minimum and maximum values. **(D)** Columella layers stained by I/KI solution in the *arf3-2* root resembled those of wild type. Seedling at 6 DAG were used. Bars = 20 μm. **(E)** QC shape was changed in ~49% *arf3-2* seedlings at 6 DAG. Bars = 20 μm. **(F)** Expression signals of *pWOX5::GFP* in *arf3-2* roots were only mildly expanded. Bars = 20 μm. **(G)**

EdU staining signals in *arf3-2* roots were indistinguishable from those in wild type. Bars = 20 μm. **(H)** Quantification of EdU staining intensity in **(G)**. VaI, Vascular Initials; CEI, Cortex/ Endodermal Initials; ELI, Epidermal/ Lateral Root Cap Initials. Wild type, n = 40; *arf3-2*, n = 40. Data are mean ± s.d. Statistical significance was determined by two-tailed Student's *t*-test.

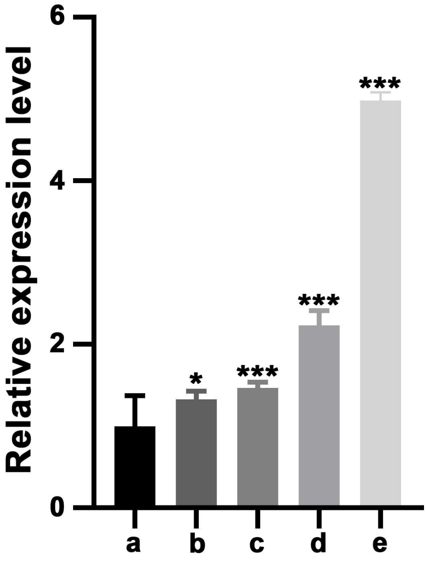

Supplementary Figure S3. *WOX5* transcript level was negatively correlated with the severity of root phenotype. (Supports Figure 2) *WOX5* transcriptional levels in individual root types of different lengths examined using qRT-PCR. Wild-type roots (a), *arf3-2* roots of normal length (b), and *arf3-2* roots with the length of ~75% (c), ~50% (d) and ~25% (e) of that of the wild type were used. Data are mean ± s.d. of three independent biological replicates. **P* < 0.05 and ****P* < 0.001 are determined by ANOVA for multiple-group comparison.

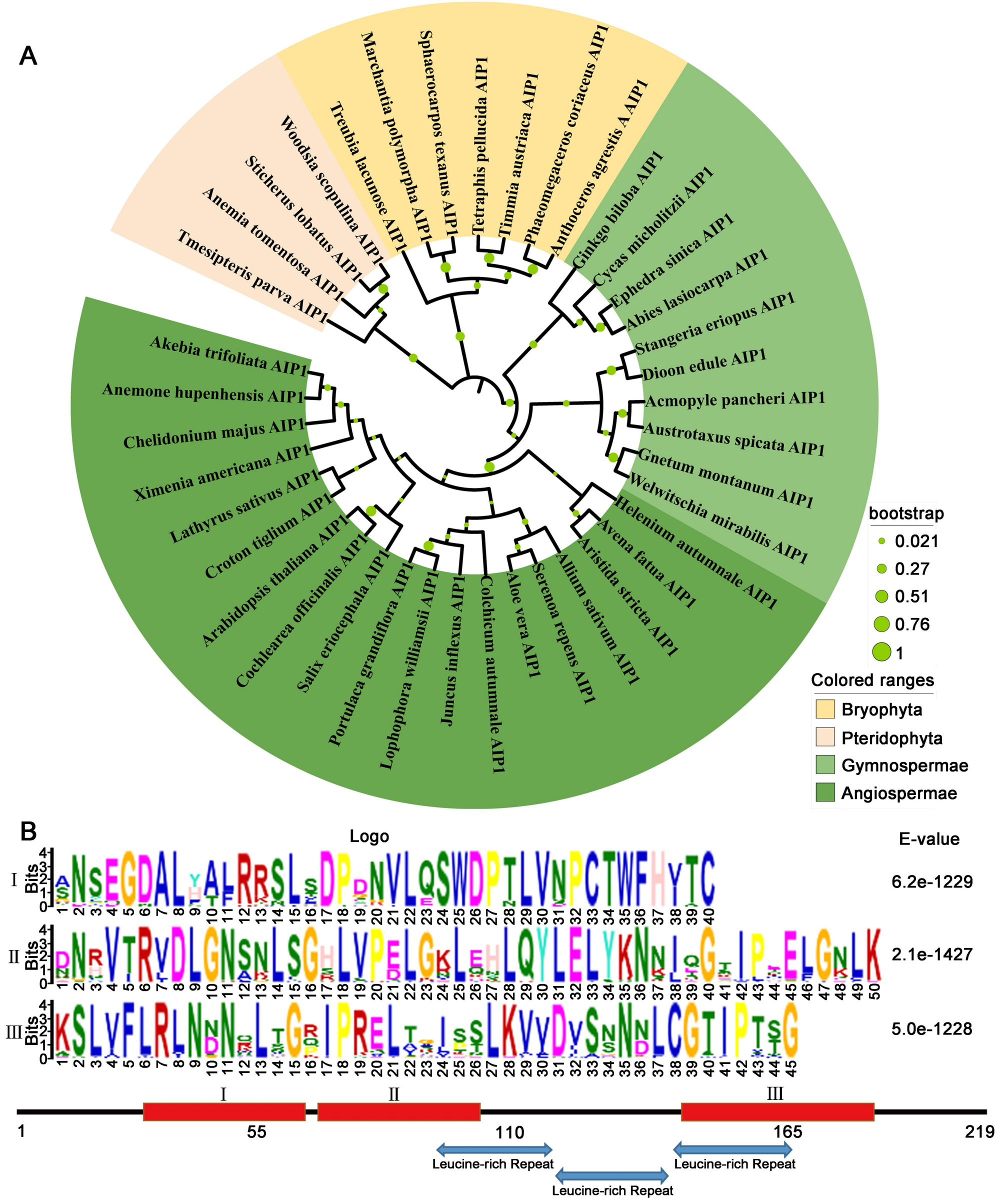

Supplementary Figure S4. Phylogenetic and Structural Analysis of AIP1. (Supports Figure 3) (A) Phylogenetic tree of the full-length protein sequences of AIP1 homologs from 40 different species representing mosses, ferns, gymnosperms and angiosperms. The phylogenetic tree was generated using the maximum-likelihood method with bootstrap values from 1000 replicates. **(B)** Conserved domains of AIP1 homologs. Top: Alignment of the conserved domains of AIP1 homologs from the same species used in **(A)**. Bottom: Schematic representation of the protein structure of *Arabidopsis* AIP1. Red boxes represent the positions of the conserved domains shown above. Double-sided arrows indicate the positions of the LRR repeats.

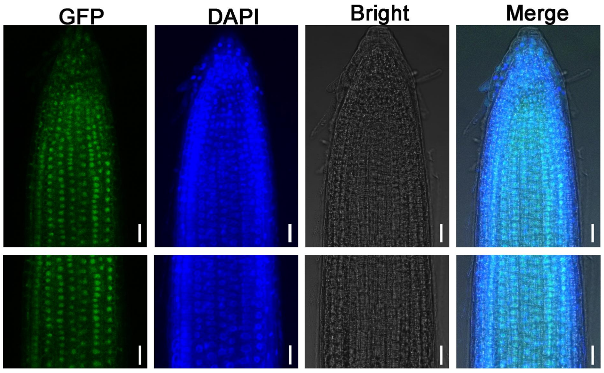

Supplementary Figure S5. AIP1 was localized in the nucleus. (Supports Figure 3) In the *35S::AIP1-GFP* root tip, GFP signals showed nuclear localization. Bars = 20 μm.

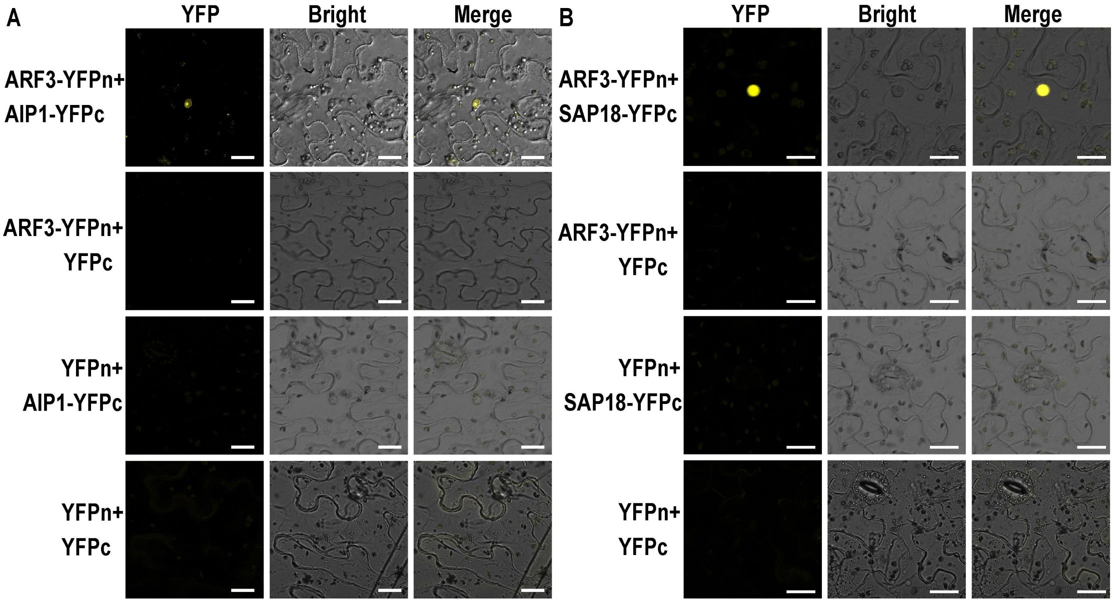

Supplementary Figure S6. Interactions between ARF3 and AIP1/SAP18 revealed by BiFC. (Supports Figure 3) (A) BiFC using YFPn-ARF3 and AIP1-YFPc illustrated reconstituted fluorescent signals. **(B)** BiFC using YFPn-ARF3 and SAP18-YFPc demonstrated reconstituted fluorescent signals. Bars = 20 μm.

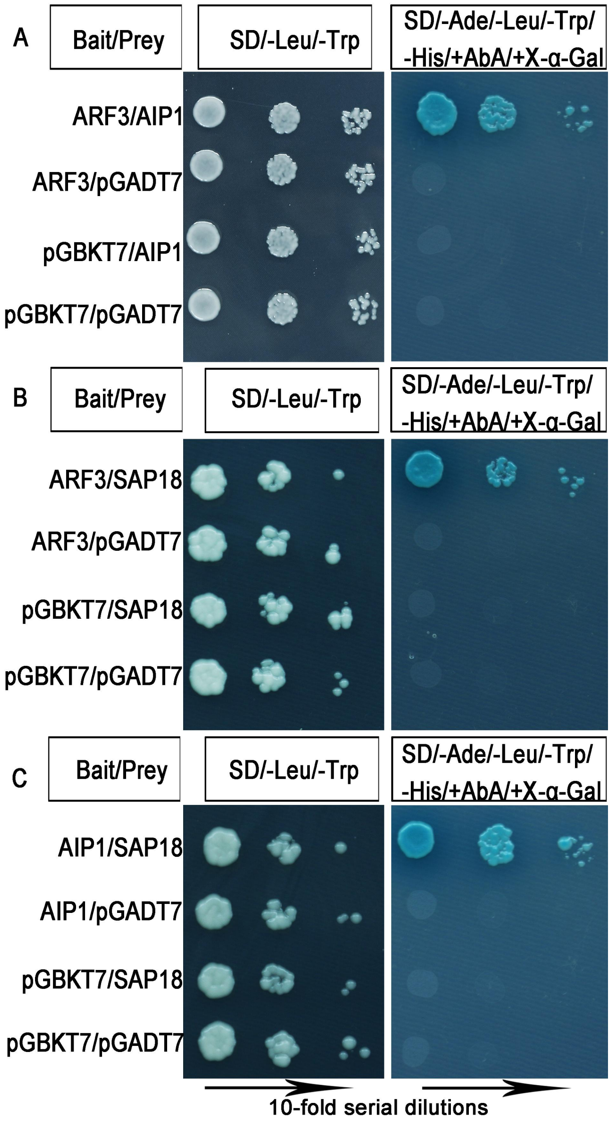

Supplementary Figure S7. Mutual interactions among ARF3, AIP1 and SAP18. (Supports Figure 3) (A) Y2H assays indicated the interaction between ARF3 and AIP1. **(B)** Y2H assays showed the interaction between ARF3 and SAP18. **(C)** Y2H assays showed the interaction between AIP1 and SAP18.

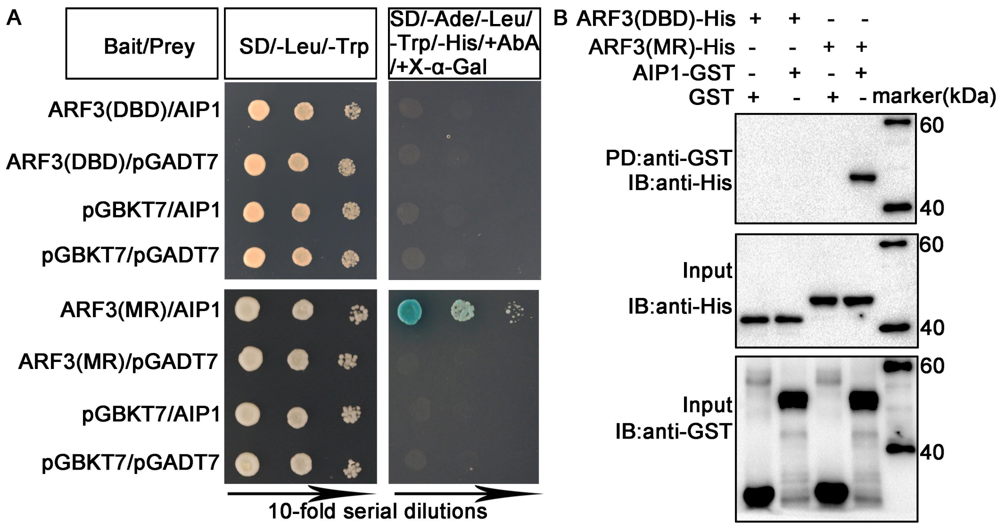

Supplementary Figure S8. AIP1 interacted with the MR domain of ARF3. (Supports Figure 3) Y2H **(A)** and pull-down **(B)** assays show that AIP1 interacted with the MR but not DBD domain of ARF3.

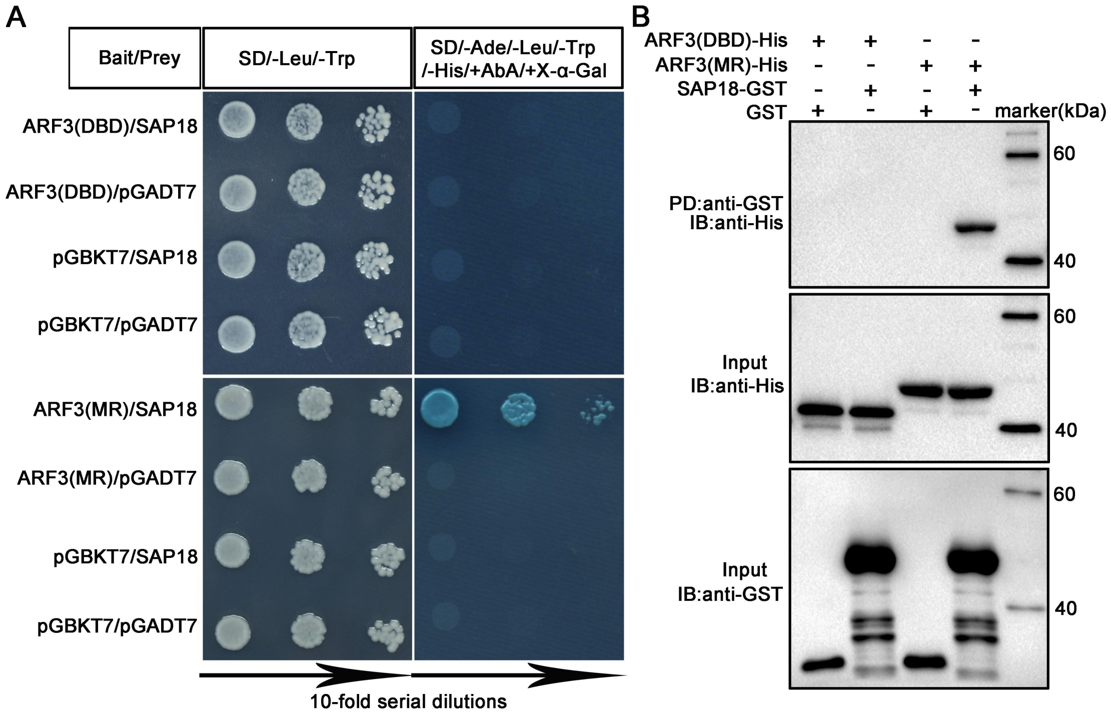

Supplementary Figure S9. SAP18 interacted with the MR domain of ARF3. (Supports Figure 3) SAP18 interacts with the MR but not DBD domain of ARF3 as illustrated by Y2H **(A)** and pull-down **(B)** assays.

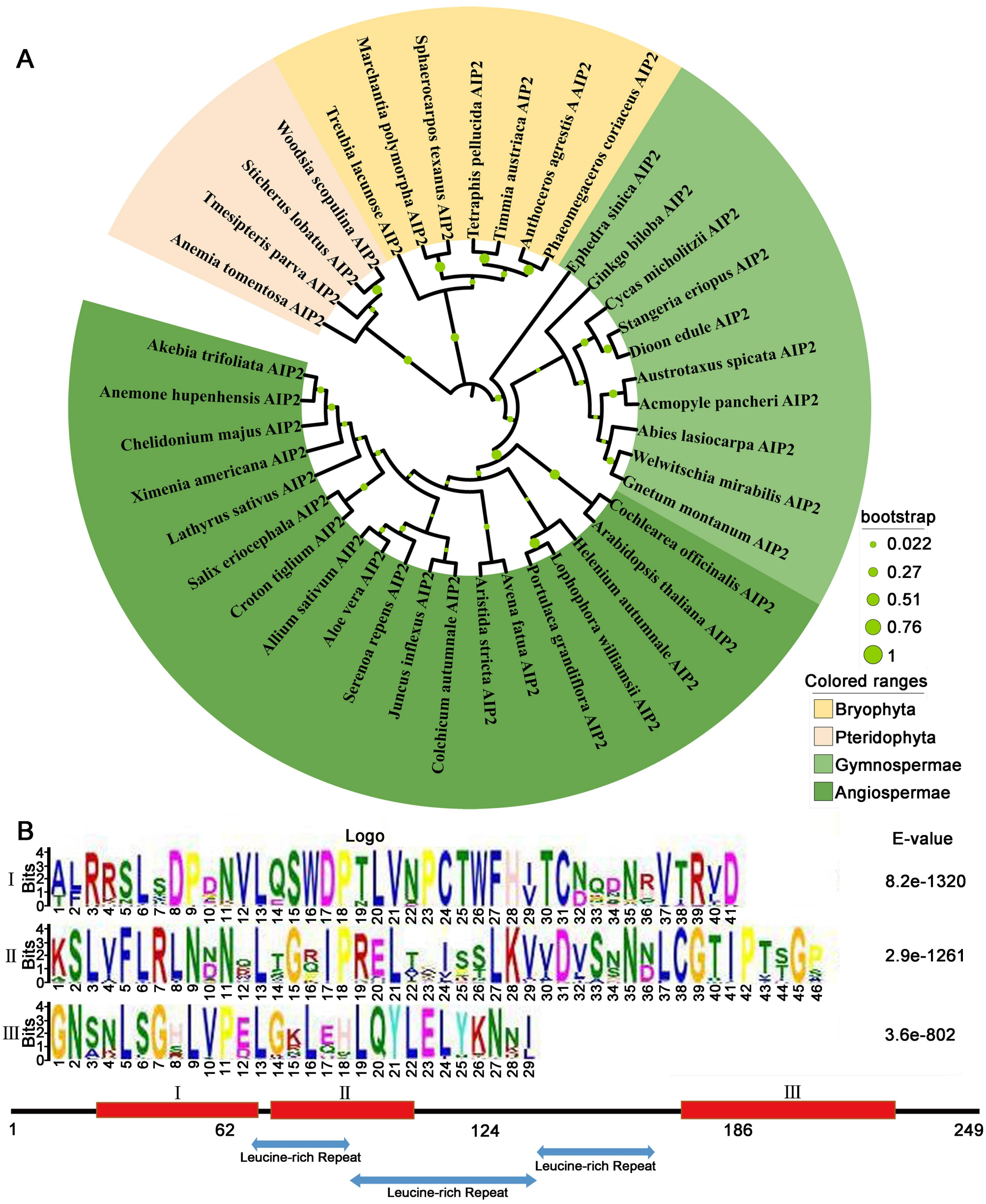

Supplementary Figure S10. Phylogenetic and structural analysis of AIP2. (Supports Figure 4) (A) Phylogenetic tree of the full-length protein sequences of AIP2 homologs from 40 different species representing mosses, ferns, gymnosperms and angiosperms. The phylogenetic tree was generated using the maximum-likelihood method with bootstrap values from 1000 replicates. **(B)** Conserved domains of AIP2 homologs. Top: Alignment of the conserved domains of AIP2 homologs from the same species used in **(A)**. Bottom: Schematic representation of the protein structure of *Arabidopsis* AIP2. Red boxes represent the positions of the conserved domains shown above. Double-sided arrows indicate the positions of the LRR repeats.

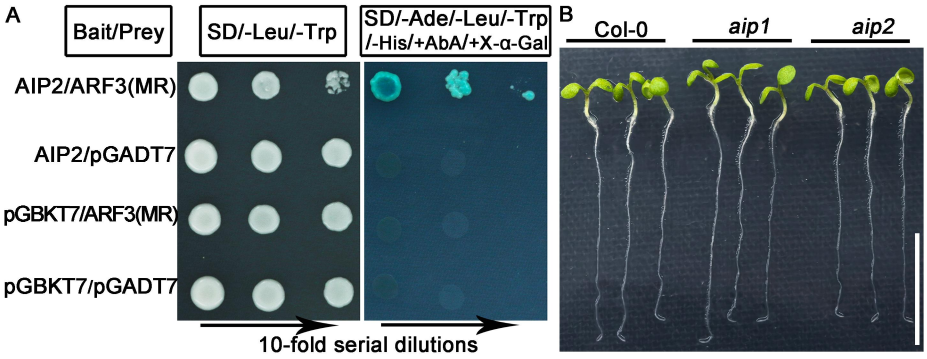

Supplementary Figure S11. AIP1 and AIP2 act redundantly in regulating root meristem maintenance. (Supports Figure 4) (A) Y2H assays demonstrated that AIP2 interacted with the MR domain of ARF3. **(B)** Roots of *aip1* and *aip2* single mutants did not show observable phenotype. Bar = 1 cm.

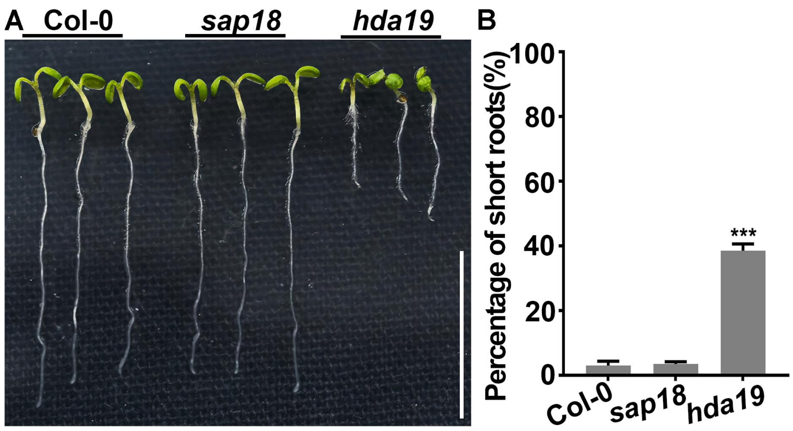

Supplementary Figure S12. Mutation in *HDA19* inhibited root elongation. (Supports Figure 4) (A) Root phenotype of wild-type *sap18* and *hda19* seedlings. Bar = 1 cm. **(B)** Percentage of short root in wild-type *sap18* and *hda19* seedlings. Data are mean ± s.d. of three independent biological replicates. ***P < 0.001 are determined by two-tailed Student's t-test.

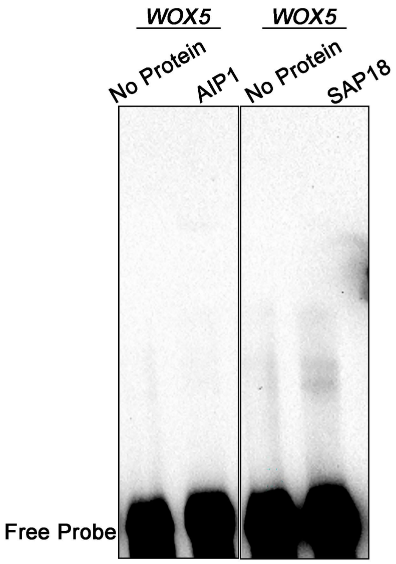

Supplementary Figure S13. EMSAs demonstrated that AIP1 and SAP18 did not directly bind the *WOX5* promoter fragments. (Supports Figure 5)

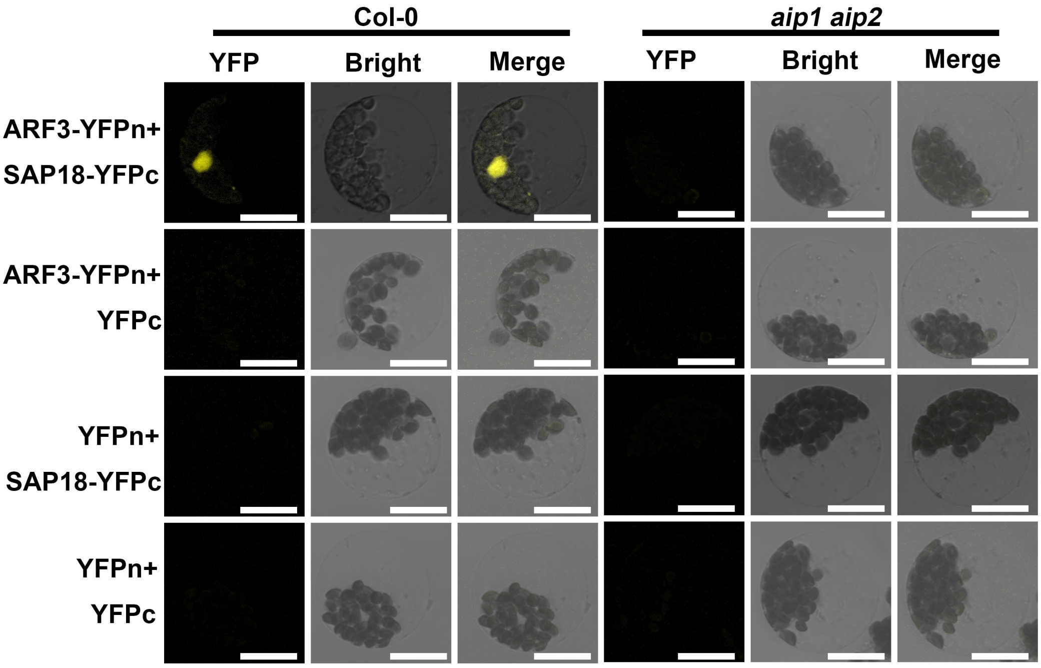

Supplementary Figure S14. Mutations in *AIP1* and *AIP2* disrupted the ARF3-SAP18 interaction. (Supports Figure 5) BiFC using YFPn-ARF3 and SAP18-YFPc demonstrated reconstituted fluorescent signals in wild-type protoplasts (left). BiFC analysis in *aip1 aip2* protoplasts using YFPn-ARF3 and SAP18-YFPc did not detect reconstituted fluorescent signals (right). Bars = 20 μm.

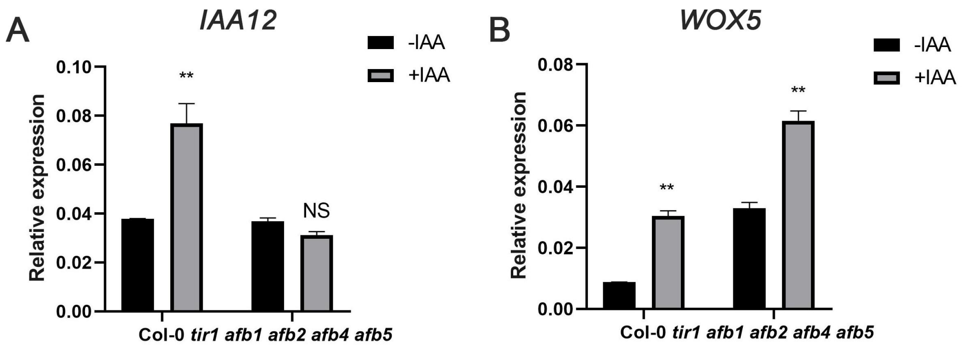

Supplementary Figure S15. The ARF3–AIP1/2–SAP18 regulated *WOX5* transcription independently of TIR1/AFB-based canonical auxin signaling. (Supports Figure 5) qRT-PCR revealed that auxin-induced transcription of *IAA12* was abolished in the *tir1 afb1 afb2 afb4 afb5* quintuple mutant **(A)**. In contrast, auxin still induced *WOX5* transcription in the quintuple mutant **(B)**. Data are mean ± s.d. of three independent biological replicates. $^{**}P < 0.01$ is determined by two-tailed Student's *t*-test.

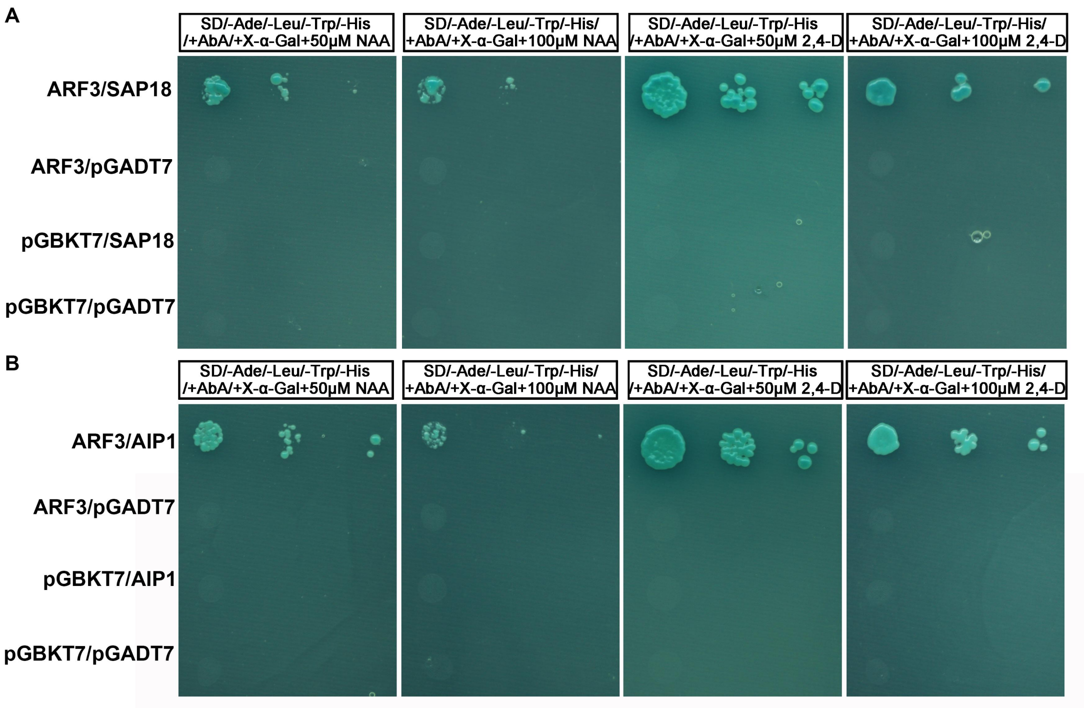

Supplementary Figure S16. Effect of NAA and 2,4-D on the ARF3-SAP18 and ARF3-AIP1 interactions. (Supports Figure 6) Exogenous NAA or 2,4-D did not eliminate the ARF3- SAP18 **(A)** and ARF3- AIP1 **(B)** interactions.

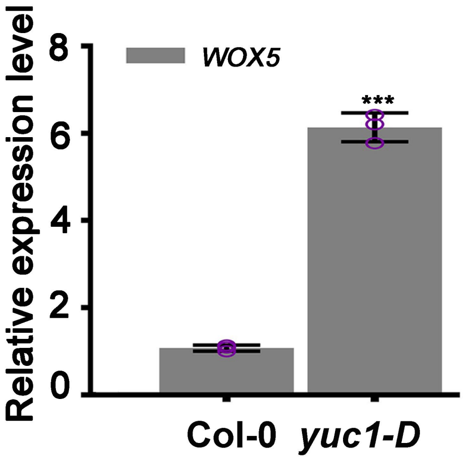

Supplementary Figure S17. Relative transcriptional level of *WOX5* was significantly increased in the *yuc1-D* mutant compared to that of wild type. (Supports Figure 7) Data are mean ± s.d. of three independent biological replicates. ***$P < 0.001$ is determined by two-tailed Student's *t*-test.

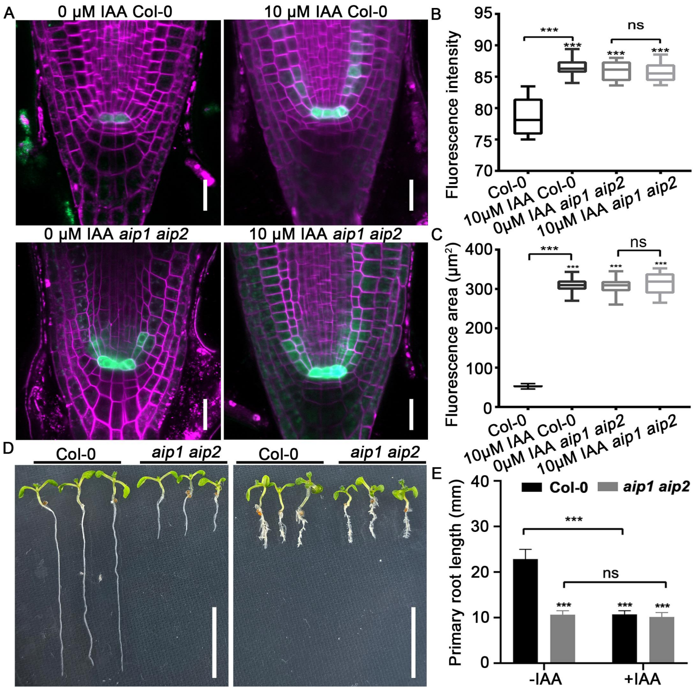

Supplementary Figure S18. The *WOX5* expression and root elongation in the *aip1 aip2* double mutant were insensitive to exogenous IAA. (Supports Figure 7) (A) *WOX5* expression patterns in wild-type and *aip1 aip2* mutant root tips in the absence and presence of exogenous IAA. Bars = 20 μm. **(B)** and **(C)**, statistics of fluorescence intensity **(B)** and area **(C)** of *pWOX5::GFP* signals in **(A)**. Wild type, n = 30; *aip1 aip2*, n = 30. Center line: median, bound of boxes: the 25th and 75th percentiles, whiskers: minimum and maximum values. **(D)** Root phenotype of wild-type and *aip1 aip2* seedlings in the absence (left) and presence (right) of exogenous IAA. Bars = 20 μm. **(E)** Statistic data of the primary root length displayed in **(D)**. In the absence of exogenous IAA, wild type, n = 40; *aip1 aip2*, n = 40. In the presence of exogenous IAA, wild type, n = 40; *aip1 aip2*, n = 40. Data are mean ± s.d. ****P* < 0.001 is determined by ANOVA for multiple-group comparison.

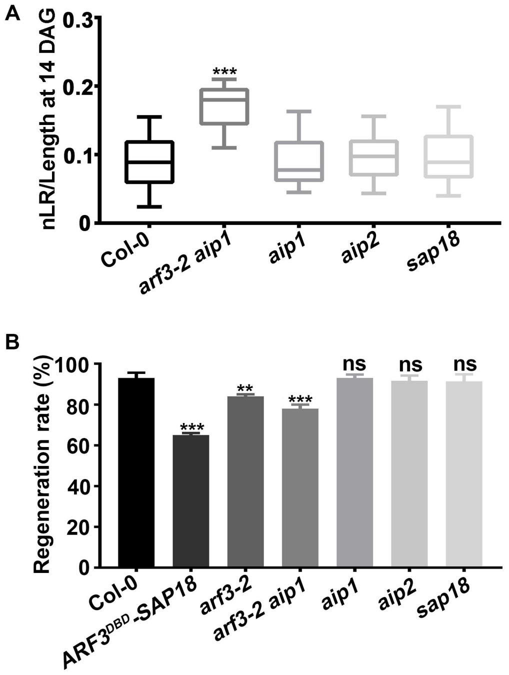

Supplementary Figure S19. Statistics of lateral root density and root-tip regeneration. (Supports Figure 8) (A) Statistics of lateral root density of wild-type, *arf3-2 aip1*, *aip1*, *aip2* and *sap18* seedlings. Wild-type, n = 53; *arf3-2 aip1*, n = 40; *aip1*, n = 44; *aip2*, n = 37; *sap18*, n = 40. Data are mean ± s.d. Center line: median, bound of boxes: the 25th and 75th percentiles, whiskers: minimum and maximum values. **(B)** Frequencies of root-tip regeneration in wild-type, *ARF3^{DBD}-SAP18*, *arf3-2*, *arf3-2 aip1*, *aip1*, *aip2* and *sap18* seedlings. Wild-type, n = 50; *ARF3^{DBD}-SAP18*, n = 50; *arf3-2*, n = 50; *arf3-2 aip1*, n = 50; *aip1*, n = 50; *aip2*, n = 50; *sap18*, n = 50. Data are mean ± s.d. of three independent biological replicates. $**P < 0.01$ and $***P < 0.001$ are determined by ANOVA for multiple-group comparison.

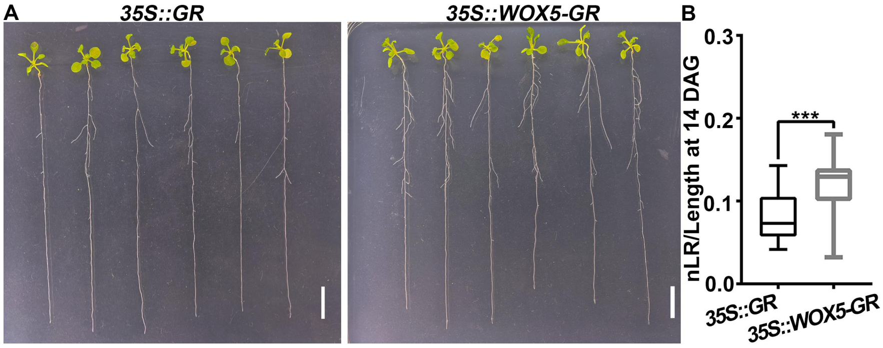

Supplementary Figure S20. Overexpressing *WOX5* or mutation in *ARF3* promoted lateral root formation. (Supports Figure 8) (A) Lateral roots in *35S::GR* control and *35S::WOX5-GR* transgenic seedlings. The seedlings were cultured in medium containing 5 μM dexamethasone. Bars = 1 cm. **(B)** Lateral root density of *35S::GR* and *35S::WOX5-GR* seedlings. *35S::GR*, n = 22; *35S::WOX5-GR*, n = 20. Data are mean ± s.d. ***$P < 0.001$ are determined by two-tailed Student's *t*-test. Center line: median, bound of boxes: the 25th and 75th percentiles, whiskers: minimum and maximum values.

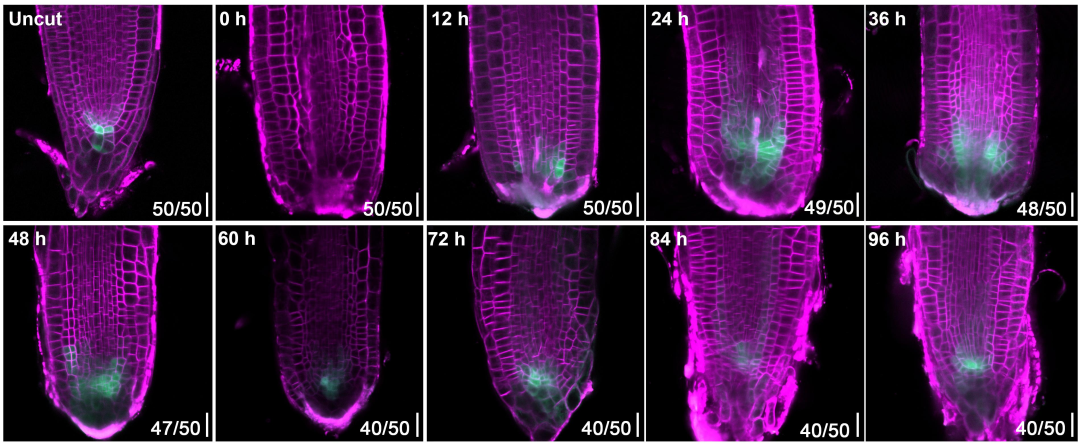

Supplementary Figure S21. Expression pattern of *pWOX5::GFP* in root-tip regeneration procedure of *arf3-2* seedlings of normal-length root. (Supports Figure 9) Bars = 20 μm. Numbers in the bottom right corner denote frequencies of the shown phenotypes.

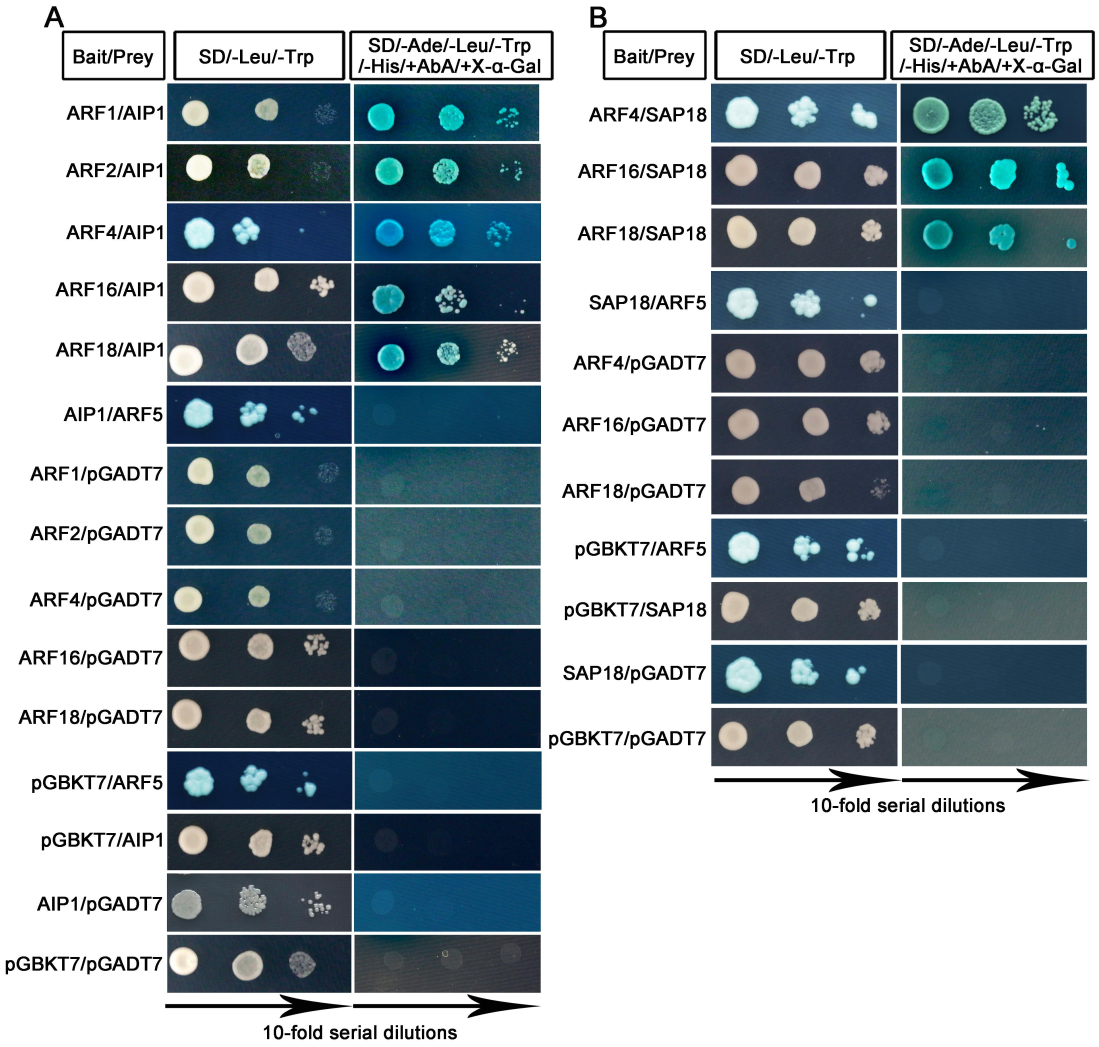

Supplementary Figure S22. AIP1 and SAP18 interacted with different repressor ARFs. (Supports Figure 10) Y2H results revealed that AIP1 **(A)** and SAP18 **(B)** interacted with different repressor ARFs, but not the activator ARF5.
