# Supplementary figures and images for: The Arabidopsis ARF3-AIP1/2-SAP18 module specifies the root stem cell niche in response to auxin
Source: Plant Cell. 2026 Apr 10;38(5):koag108. doi: 10.1093/plcell/koag108 (PMC13221652; doi:10.1093/plcell/koag108)

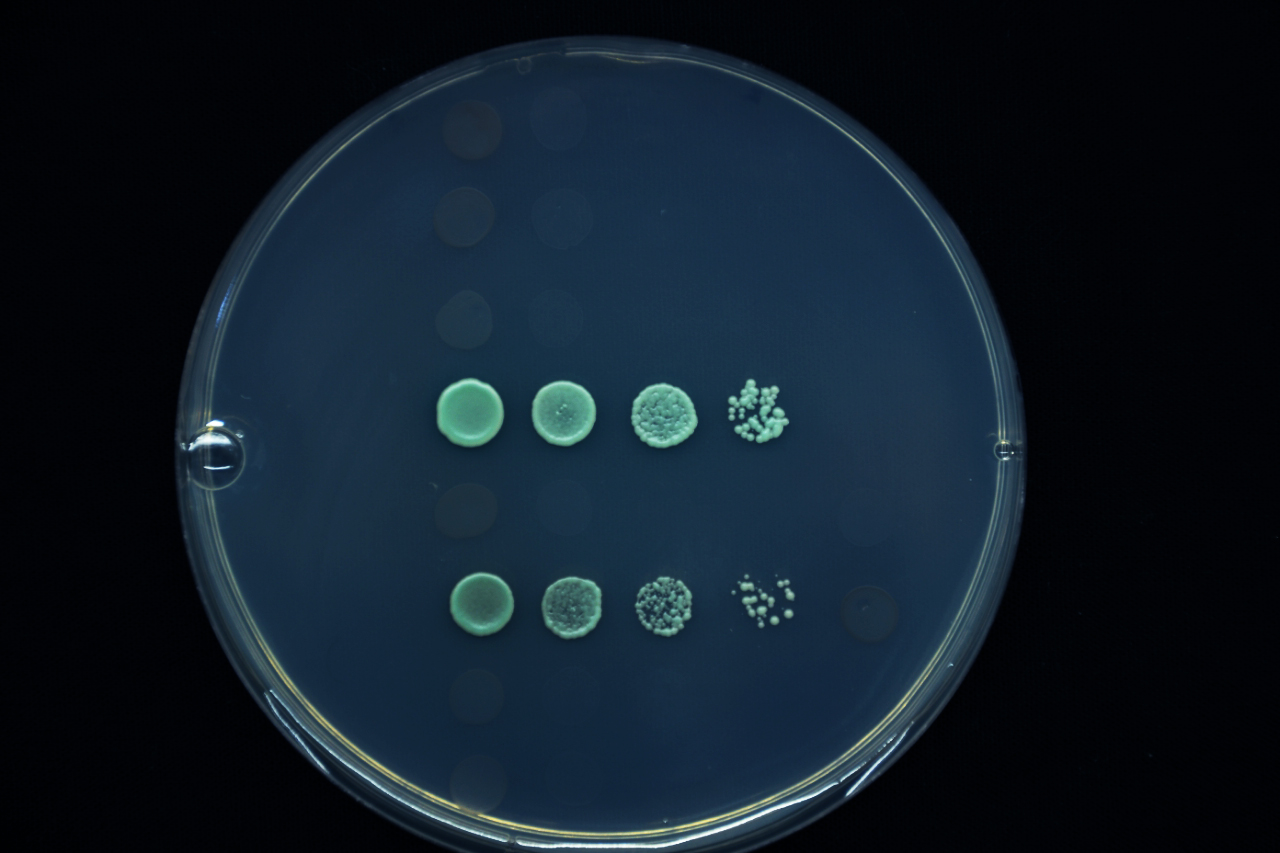

Supplement: koag108_Supplementary_Data [file koag108_supplementary_data.zip › Image source files of Supplementary Figures 22 right, 1st line, ARF4-SAP18.jpg]

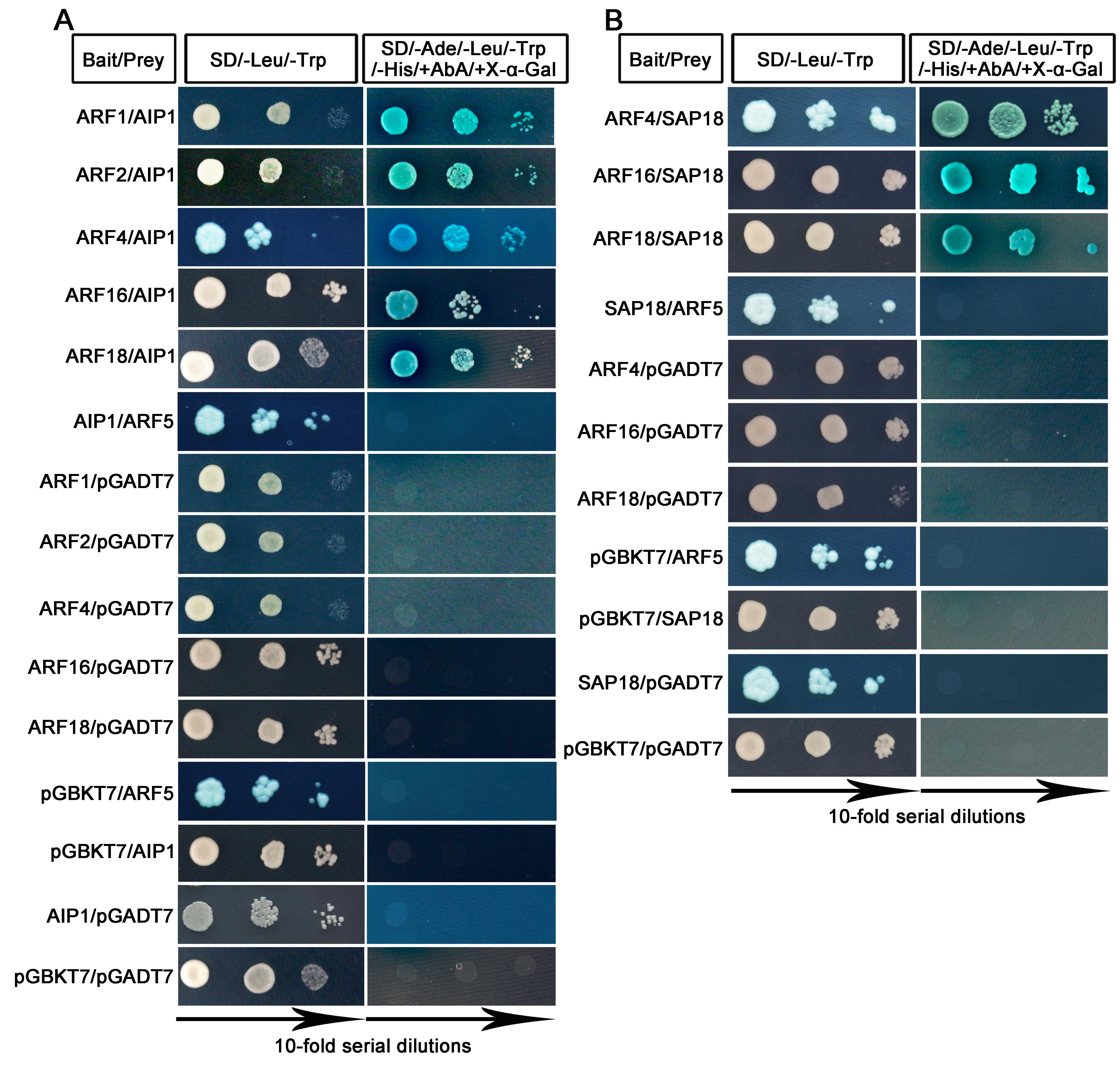

Supplement: koag108_Supplementary_Data [file koag108_supplementary_data.zip › Revised Supplementary Figure S22.tif]

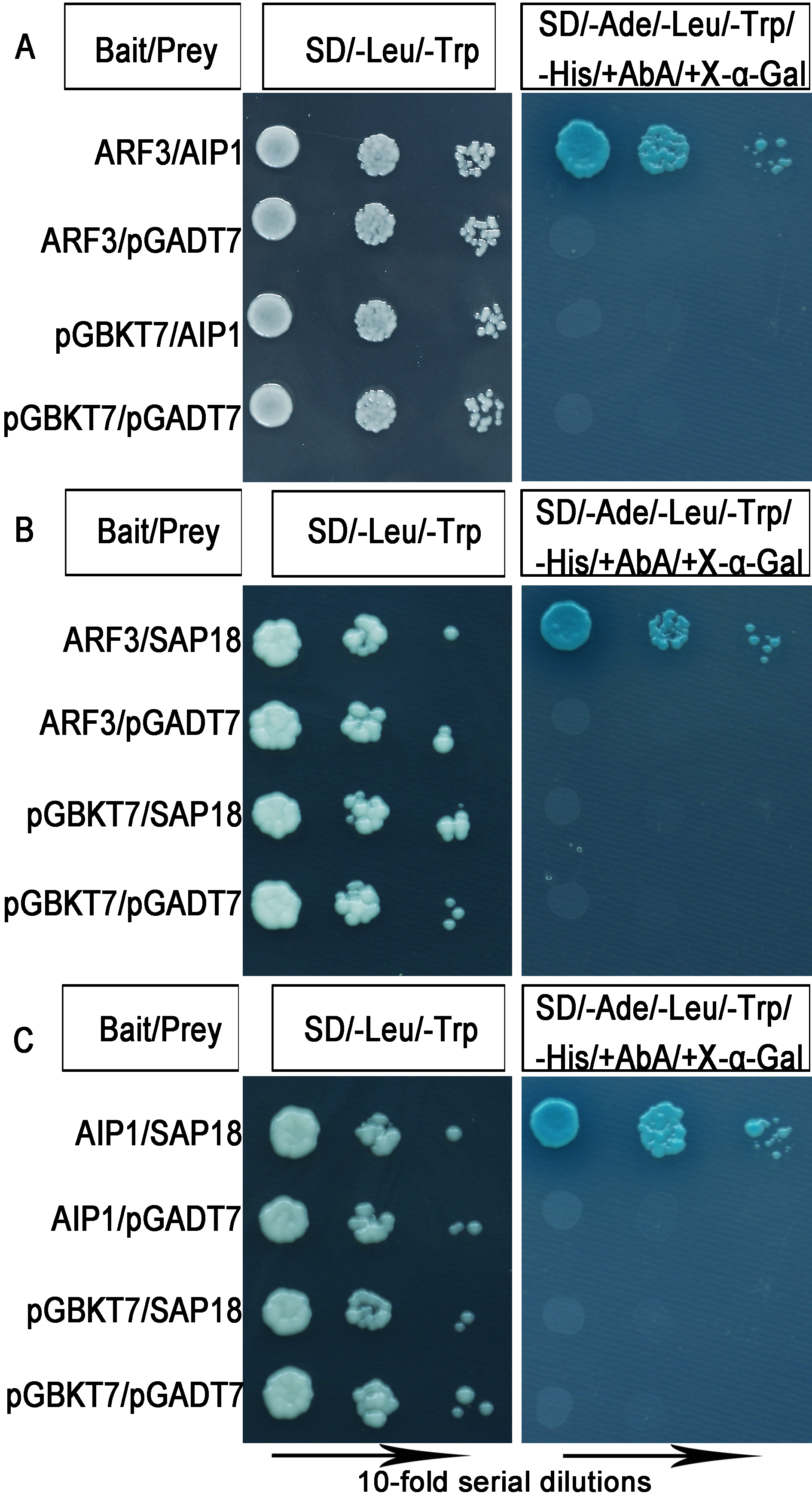

Supplement: koag108_Supplementary_Data [file koag108_supplementary_data.zip › Revised Supplementary Figure S7.tif]

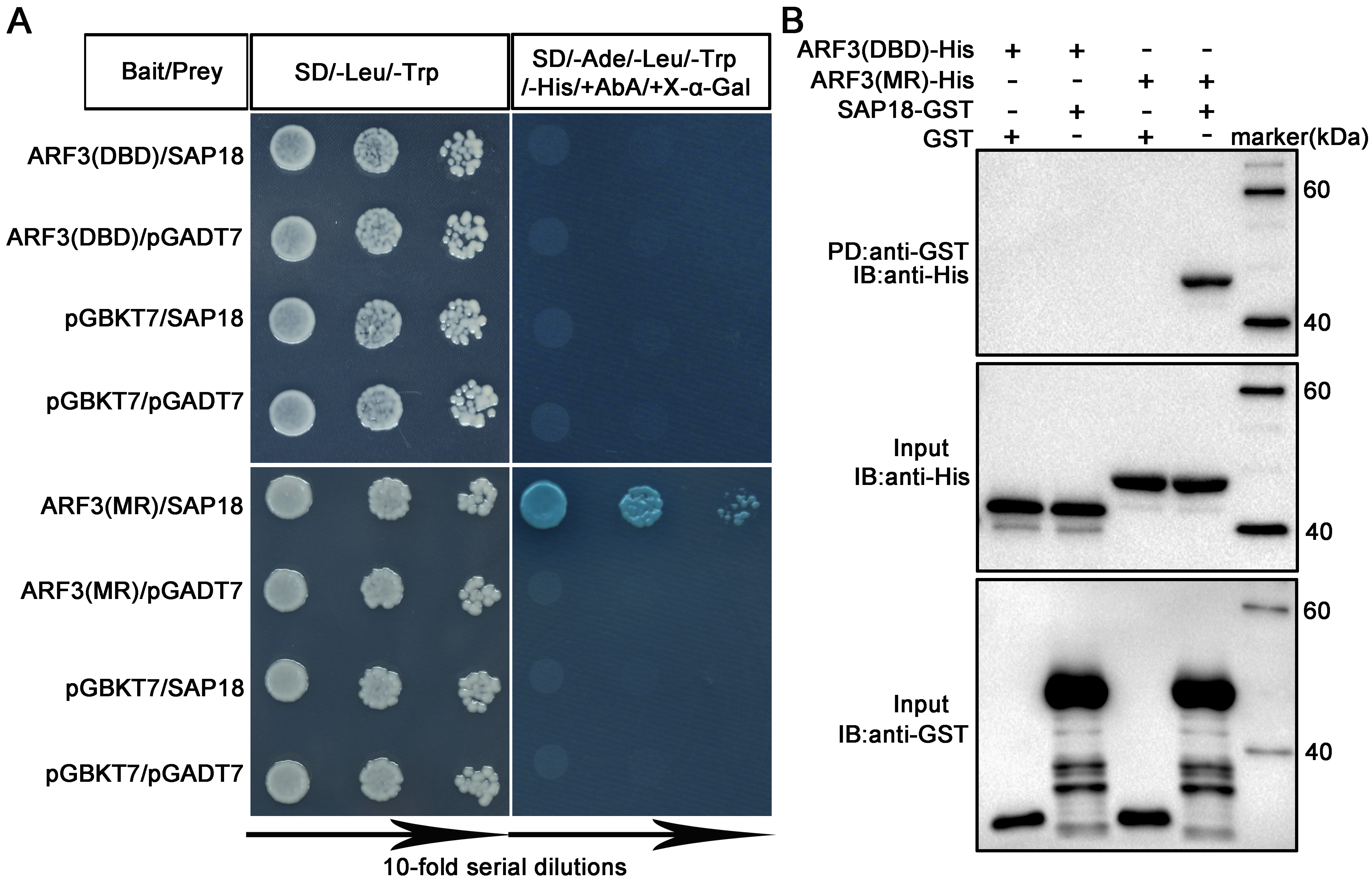

Supplement: koag108_Supplementary_Data [file koag108_supplementary_data.zip › Revised Supplementary Figure S9.tif]

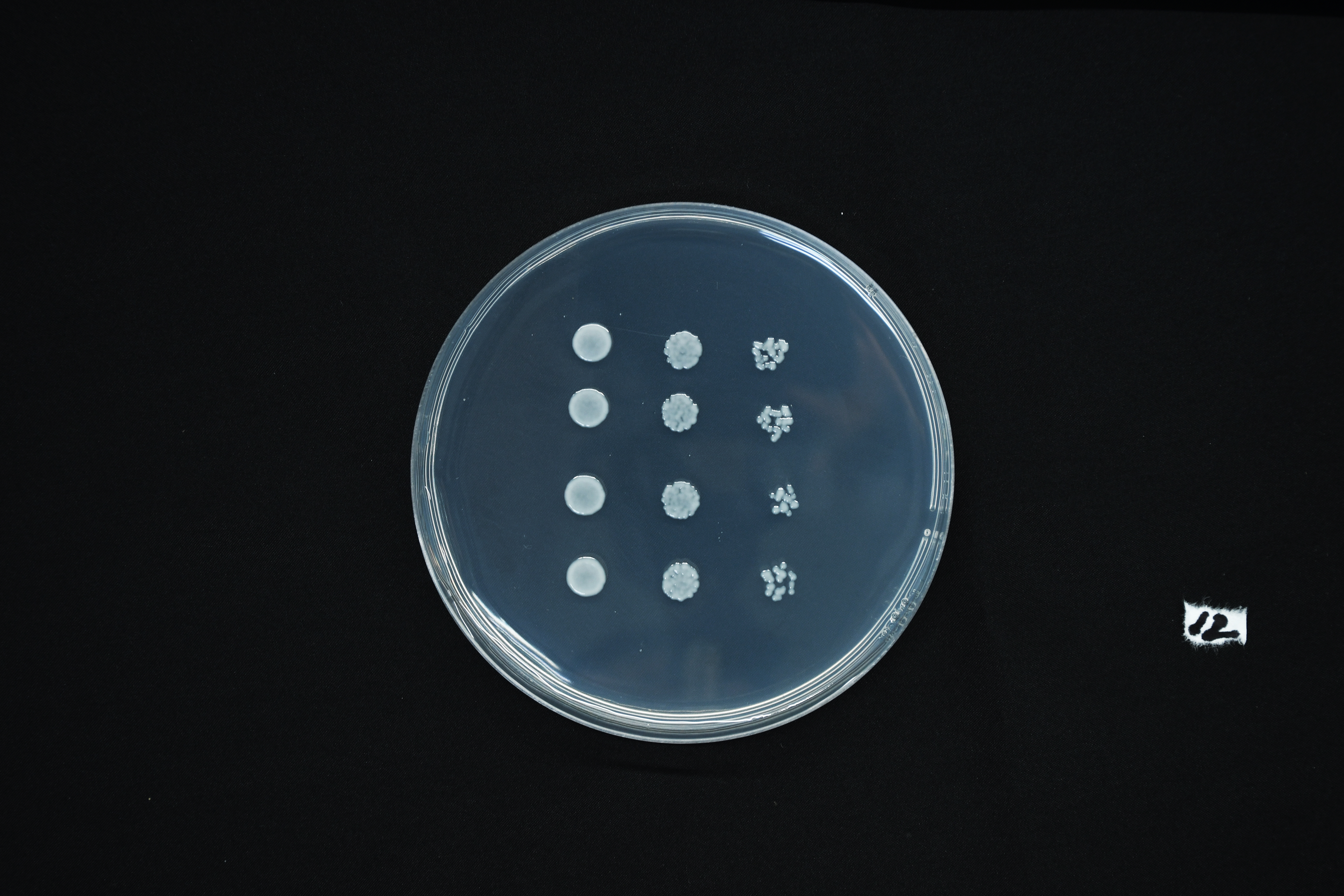

Supplement: koag108_Supplementary_Data [file koag108_supplementary_data.zip › Supplementary Figure S7A left.jpg]

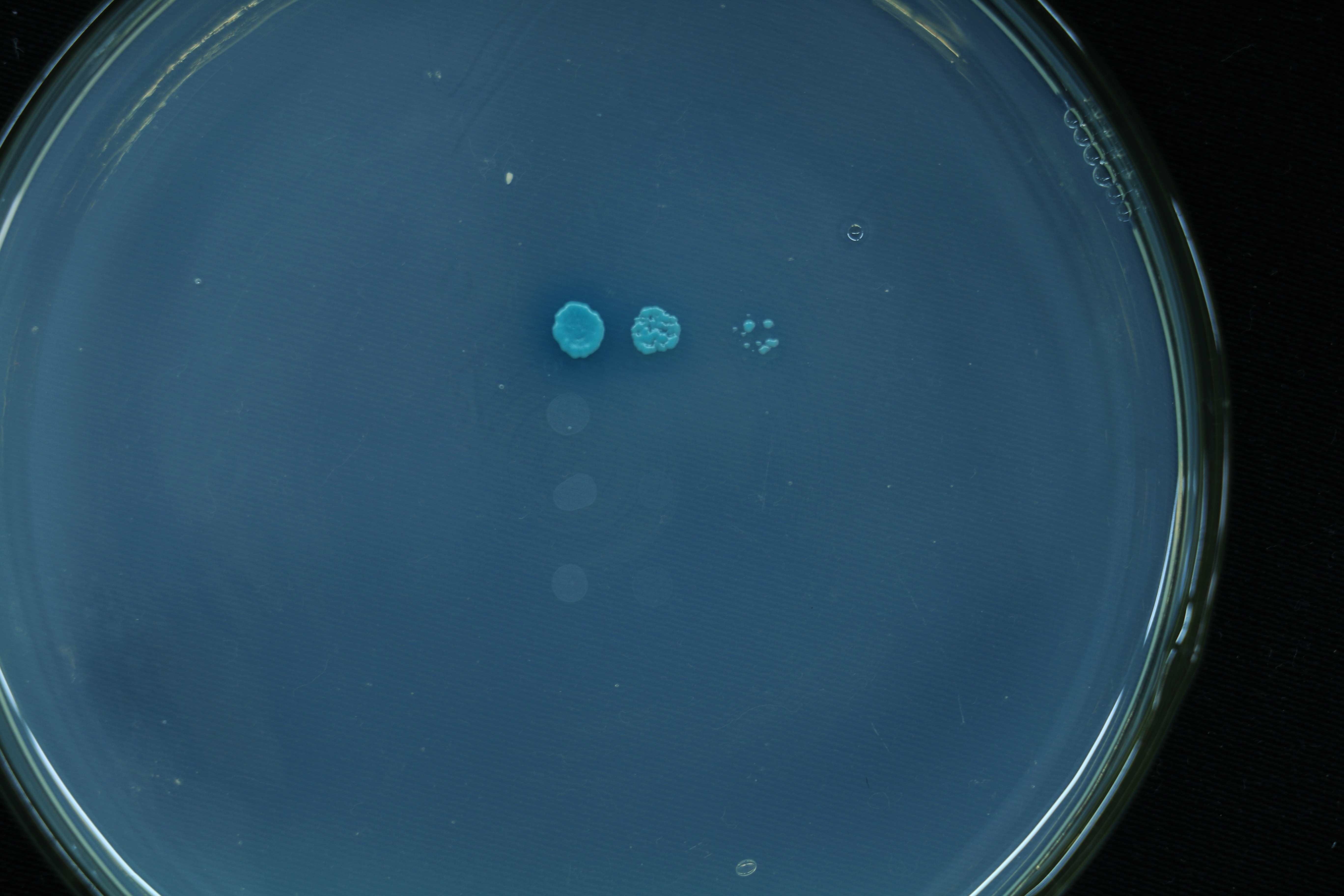

Supplement: koag108_Supplementary_Data [file koag108_supplementary_data.zip › Supplementary Figure S7A right.jpg]

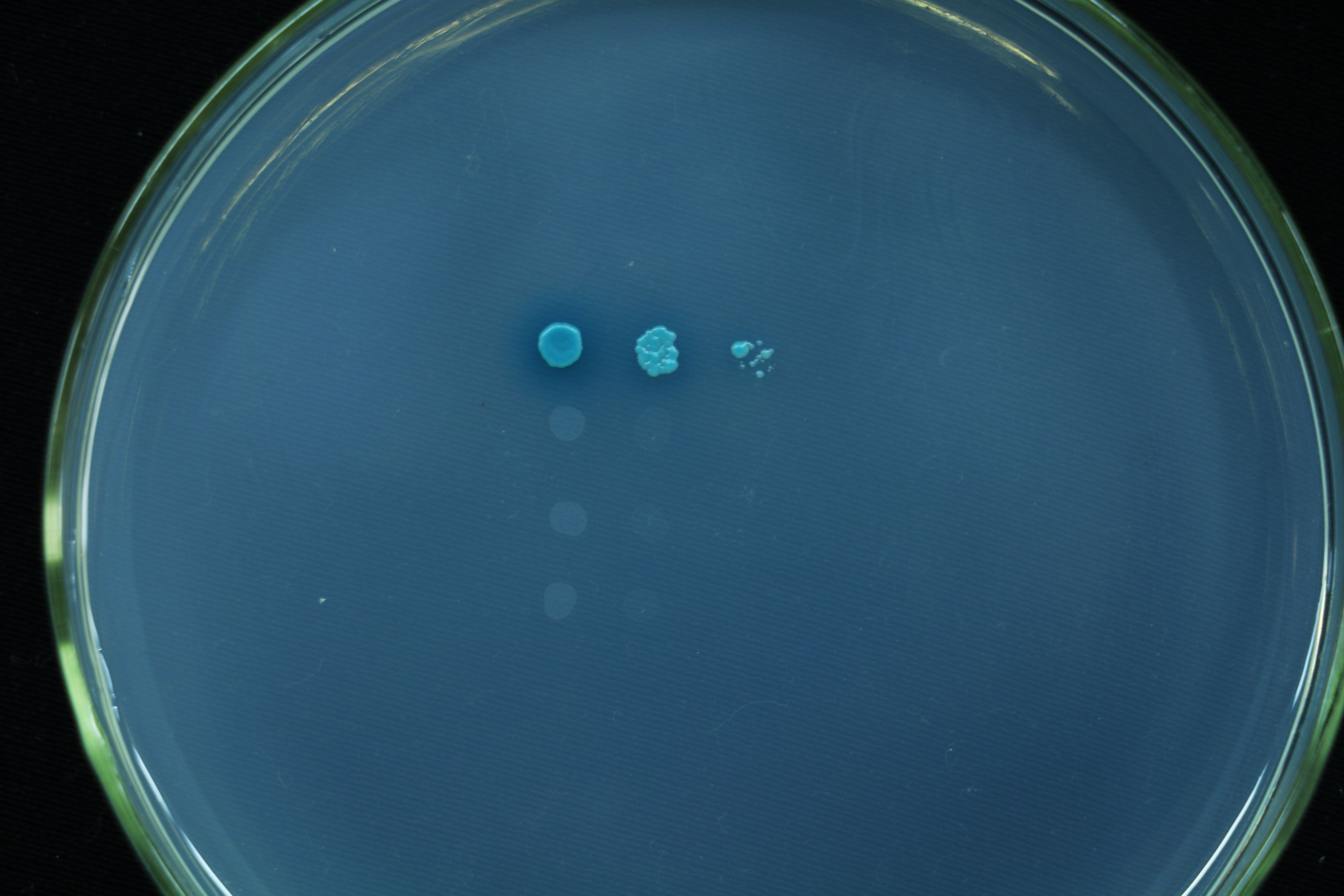

Supplement: koag108_Supplementary_Data [file koag108_supplementary_data.zip › Supplementary Figure S7C right.jpg]

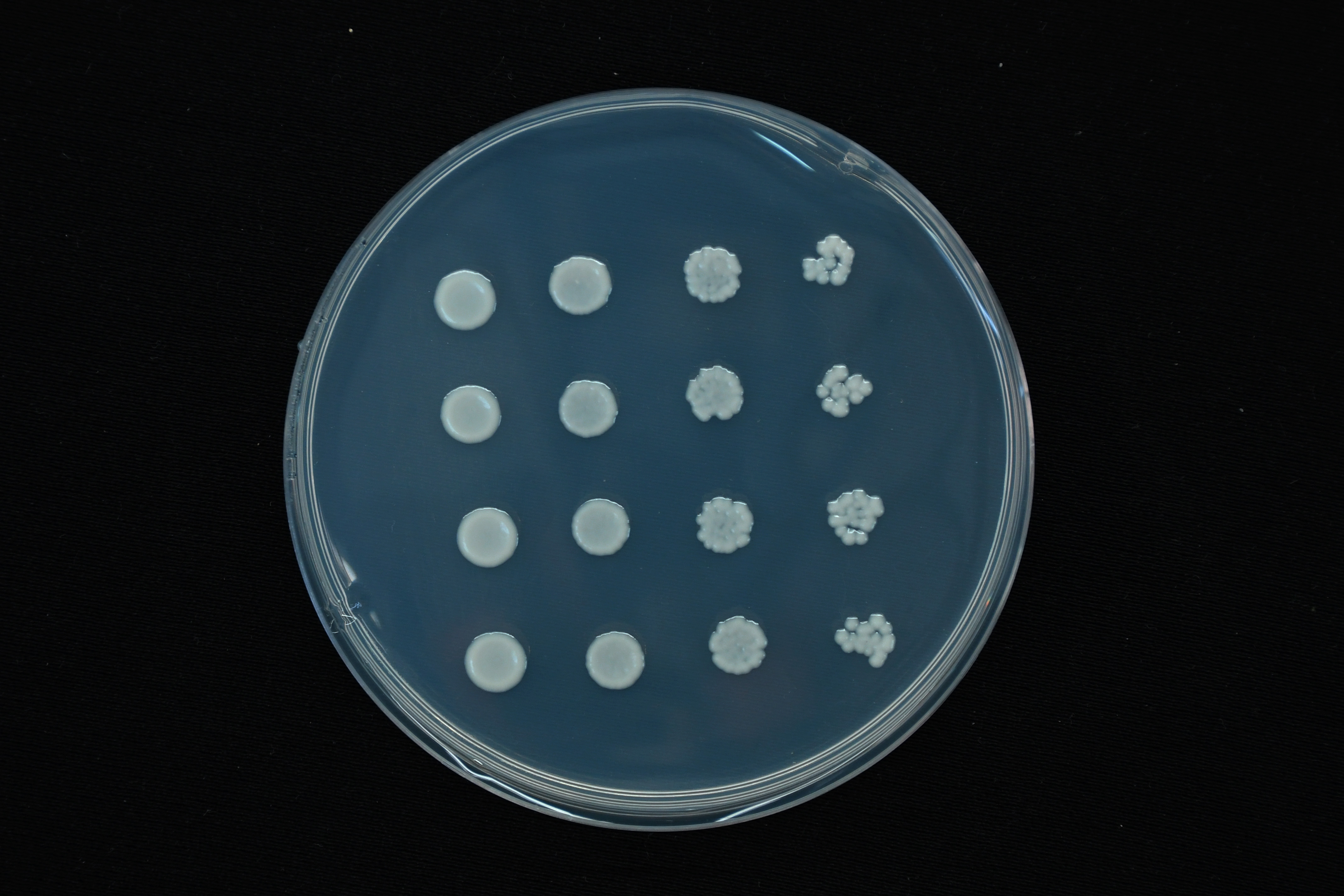

Supplement: koag108_Supplementary_Data [file koag108_supplementary_data.zip › Supplementary Figure S9A left lower panel.jpg]

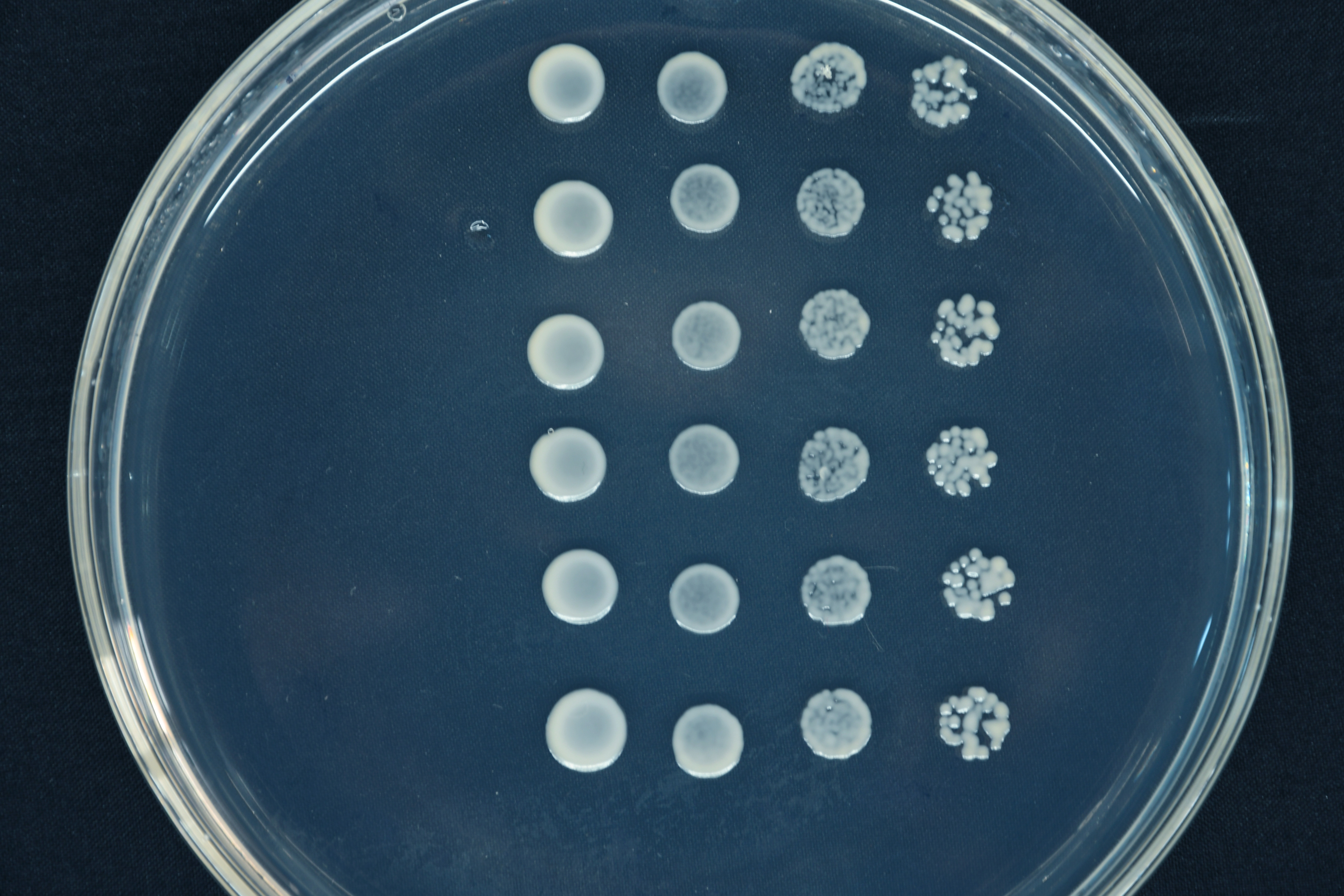

Supplement: koag108_Supplementary_Data [file koag108_supplementary_data.zip › Supplementary Figure S9A left upper panel.jpg]

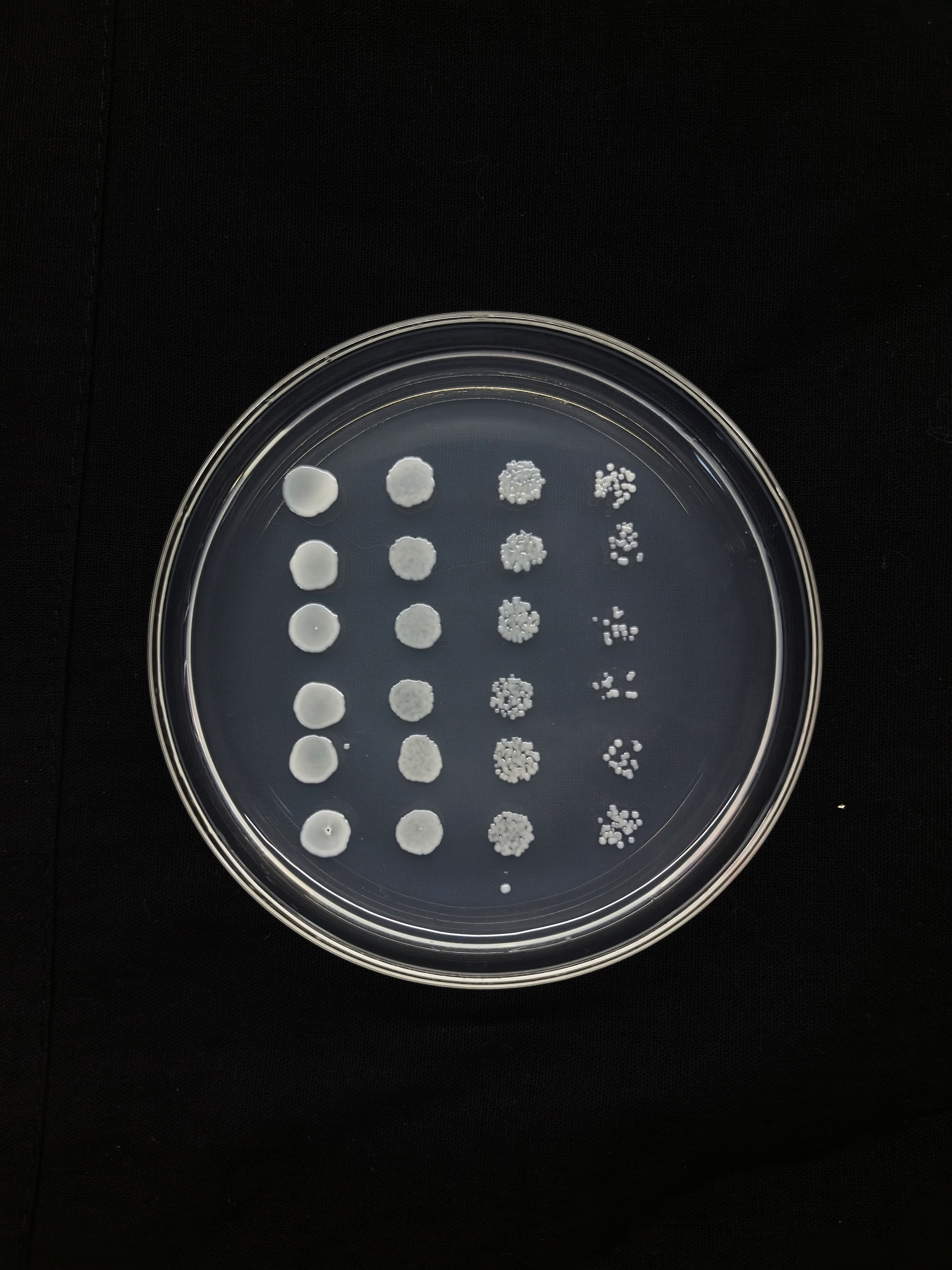

Supplement: koag108_Supplementary_Data [file koag108_supplementary_data.zip › Supplementary Figures 22 left, 14th line, AIP1pGADT7.jpg]
